# Defining and classifying adverse events following joint manipulation and mobilization: An international e-Delphi study and focus groups

**DOI:** 10.1371/journal.pone.0334151

**Published:** 2025-11-17

**Authors:** Martha Funabashi, Lindsay M Gorrell, Katherine A Pohlman, Andrea Bergna, Nicola R Heneghan, on behalf of the AdEMMo Group

**Affiliations:** 1 Division of Research and Innovation, Canadian Memorial Chiropractic College, Toronto, Canada; 2 Department of Chiropractic, Université du Québec à Trois-Rivières, Trois-Rivières, Canada; 3 Integrative Spinal Research Group, Department of Chiropractic Medicine, University Hospital Balgrist, Zurich, Switzerland; 4 University of Zurich, Zurich, Switzerland; 5 Research Center, Parker University, Dallas, Texas, United States of America; 6 Research Department, SOMA Istituto Osteopatia Milano, Milan, Italy; 7 AISO-Associazione Italiana Scuole di Osteopatia, Pescara, Italy; 8 School of Sport, Exercise & Rehabilitation Sciences, University of Birmingham, Birmingham, United Kingdom; IST: Universidade de Lisboa Instituto Superior Tecnico, PORTUGAL

## Abstract

Spinal and peripheral joint manipulation (MAN) and mobilization (MOB) are widely used for managing musculoskeletal conditions. Although adverse events (AE) have been reported following these interventions, there is no universally accepted definition and classification system. This study aimed to establish an inter-professional and international standardized definition and severity classification for AE following MAN and MOB. This sequential mixed-methods study included an electronic Delphi process (e-Delphi) followed by focus groups. Inter-professional and international expert stakeholders participated in 3 e-Delphi rounds: Round 1 included open-ended questions on participants’ working AE definition and severity classification; Round 2, level of agreement with statements generated from Round 1 and a previous scoping review; and Round 3, level of agreement with statements achieving consensus in Round 2. Focus groups explored e-Delphi findings. Consensus was reached for severity categories (i.e., mild, moderate, severe and catastrophic) and on 2 domains to differentiate these categories (i.e., symptom intensity and impact on patient). Consensus was not reached for a standardized AE definition following MAN and MOB. Focus group discussions centered on “unfavourable”, “unexpected” and “undesired” terms and differences between “serious” and “catastrophic” severity classification categories. Findings contribute to advancing patient safety and AE knowledge across professions and informing further safety research and practice.

## Introduction

Spinal and peripheral joint manipulation and mobilization are interventions commonly used by many healthcare professionals (such as physiotherapists, chiropractors, osteopaths, etc.) to manage musculoskeletal conditions, including spinal pain [1,2]. Manipulation techniques (MAN) involve the manual application of a dynamic high-velocity, low-amplitude force; whereas mobilization techniques (MOB) involve the application of a cyclic low-velocity and variable amplitude manual force [3].

There is increasing evidence supporting the effectiveness of these interventions to reduce pain and improve function in patients with musculoskeletal conditions with MAN and MOB both recommended in numerous clinical practice guidelines [4–8]. Despite this, research focused on the safety of MAN and MOB remains in its infancy and limited in both scope and depth, making it challenging to fully assess the risk-benefit profile of these interventions.

In all healthcare professions, patient safety is a priority, with emphasis on minimizing preventable and/or unexpected adverse events (AE) during or following any clinical intervention, including MAN and MOB [9,10]. However, within the field of MAN and MOB, efforts to reduce AE have been hindered by a number of factors, including: i) heterogeneity in AE definitions; their nuanced wording and classification; ii) a lack of reporting systems that accurately and consistently collect safety information; iii) long periods of time between patient visits; and iv) a lack of coordination of care among multiple providers [9,11–14]. Consequently, addressing these factors and collecting relevant AE data in a standardized way is fundamental to improve patient safety [15].

This is challenging considering the spectrum of AE reported in the literature following MAN and MOB, ranging from frequent and expected minor AE (e.g., mild discomfort and increased muscle soreness after treatment) to rare and serious AE (e.g., cervical artery dissection and subsequent stroke, or death) [12,16–18]. Furthermore, a recent scoping review of the literature, encompassing several healthcare professions that use MAN and MOB, reported that a wide variety of terms and definitions are used to describe AE and, similarly, several systems are used to classify AEs [19]. Authors concluded there was no one standardized definition or classification system for reporting AE that was commonly used. This lack of inter-professional uniformity globally not only hinders the possibility of accurately quantifying AE incidence rates, but also impedes advancement in patient safety initiatives and strategies. Additionally, the heterogeneity in AE definitions also precludes the accurate reporting of AEs and hinders effective communication among healthcare professionals, which can negatively impact patient outcomes. Therefore, to address this need, this study aimed to establish an inter-professional and international standardized definition and severity classification for AE following spinal and peripheral joint manipulation and mobilization, within an adult population with musculoskeletal conditions.

## Methods

### Design

**Fig 1:**
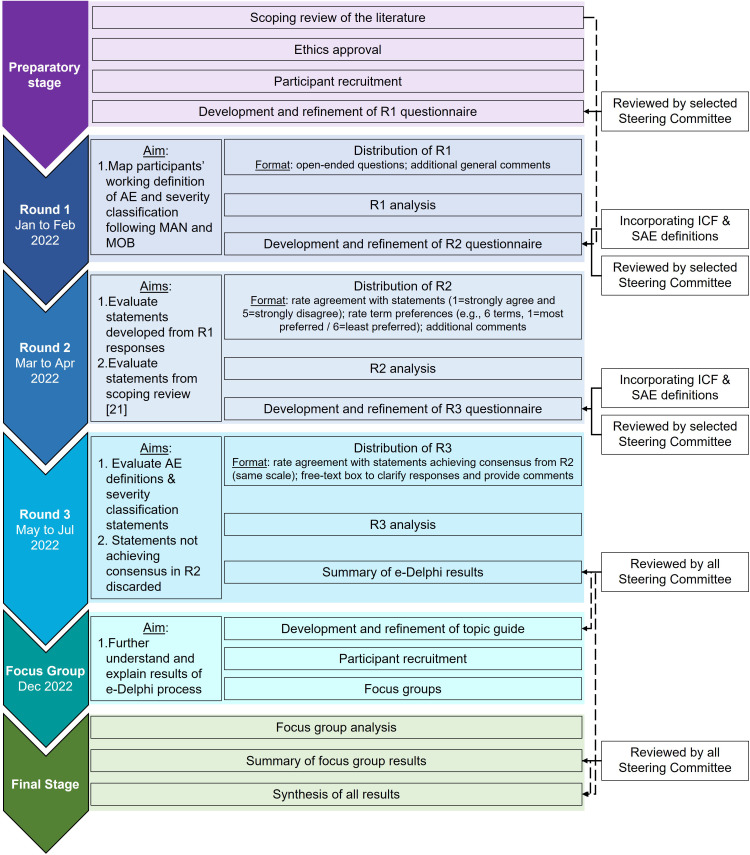
Delphi study procedures.

A sequential explanatory mixed-methods design consisting of: 1) an electronic Delphi process (e-Delphi process); and 2) virtual focus groups was used. The e-Delphi (January to July 2022) consisted of three rounds of questionnaires collected electronically using the Research Electronic Data Capture system (REDCap®) platform [20,21]. To help further understand and explain the results of the e-Delphi process, 3 focus groups were conducted in December 2022 (Fig 1).

A detailed rationale and justification for this design was previously described [22]. Briefly, the e-Delphi method was chosen as it addresses barriers from other consensus approaches, such as nominal group technique. Specifically, by overcoming differences in geographical location, time zones, and participants schedules, this method allowed for the participation of experts globally and without limits to specific participant groups.

All parts of this study were in accordance with the ethical standards, guidelines and regulations of the responsible committee, with the Helsinki Declaration and approved by the Canadian Memorial Chiropractic College Research Ethics Board (CMCC REB# 2103B01). All participants signed an electronic informed consent form prior to participation. The AdEMMo group (*full name of members in acknowledgment section*), comprised of 14 inter-professional and international members with expertise in patient safety, research methodology, and MAN/MOB, acted as a steering committee. Given their knowledge and expertise in the area, they were divided into two sub-committees that provided their opinions by either: i) participating in each e-Delphi round as a respondent (CB, DC, ES, LC, MOB, MH, PaD, SV, SL); or ii) reviewing results at each round, providing feedback on questionnaire development, and approving questionnaires to be sent to participants (AG, PeD, RE, SM, SS) [22]. The AdEMMo group included a patient representative who was a member in the sub-committee reviewing rounds results and questionnaires ensuring patients’ perspectives were taken into consideration. The e-Delphi is reported in accordance with the “Guidance on Conducting and Reporting Delphi Studies (CREDES)” [S1 Table] [23]. Similarly, the qualitative portions of this study (round 1 of the e-Delphi process and focus groups) are reported in accordance with the “Standards for Reporting Qualitative Research (SRQR)” [S2 Table] [24].

### Procedure

#### e-Delphi process.

The e-Delphi process consisted of three rounds of questionnaires (Fig 1). A detailed description of each round was previously provided [22]. Briefly, round 1 questionnaire consisted of open-ended questions of participants’ current understanding of definition and severity classification of AEs following joint MAN and MOB. Round 2 included statements developed based on round 1 responses and findings from a previous scoping review of the literature mapping the definition and classification of AEs previous studies investigating joint MAN and MOB used [19]. Round 3 included feedback from round 2 using descriptive statistics to promote participant reflection and the questionnaire consisted of statements from round 2 that reached consensus for further agreement. Definitions identified in the scoping review of the literature were only presented to participants in round 2 to avoid anchoring bias, encourage independent, discipline-specific contributions and ensure that the resulting consensus was not influenced by the literature or the researchers’ preconceptions [23]. Questionnaires were reviewed and piloted by (expert methodologist) members of the AdEMMo group and edited based on their feedback [S1 File]. All participants were invited to respond to all rounds to provide them with the opportunity to continue their involvement even if they were unable to complete previous rounds. Each round remained active for 6 weeks with email reminders sent in weeks 1, 3 and 5 [22].

#### Focus groups.

A topic guide was developed based on e-Delphi round 3 results with questions and prompts designed to encourage participant discussion [S3 Table]. A pilot focus group was conducted with colleagues from the investigative team that were not involved with the study to simulate the discussions as well as test and further refine the topic guide. Following the pilot focus group, modifications to the topic guide were made, including re-wording and re-ordering of questions.

Three virtual focus groups were conducted using an online platform (Zoom Video Communications, Inc., CA, USA). Conducting three focus groups would allow for thematic saturation and cross-group comparison, while accounting for practical constraints related to group size and participant availability across time zones. A summary of the e-Delphi results and discussion topics were circulated to participants *a priori* in pdf format [S2 File]. Each focus group was scheduled for 120 minutes and was digitally recorded. The facilitator (MF) encouraged all participants to actively contribute to the discussion to ensure that everyone had the opportunity to voice their opinions and thoughts on all topics. The moderator (NH) kept track of time to ensure all topics were discussed and monitored the chat box for written comments. In addition to notes taken by both the facilitator and moderator, each focus group was transcribed verbatim. Participants in each session had the opportunity to review their transcripts and add further comments prior to data analysis.

### Participant eligibility and sample

#### e-Delphi process.

Inclusion and exclusion criteria and participant recruitment were previously detailed [22]. Briefly, participants were adults (≥ 18 years old) with a high level of knowledge within the area of patient safety and AE related to MAN and MOB (Table 1). They were identified and recruited through social media, emails to professional networks, and authors of studies included in the previous scoping review [19]. Care was taken to ensure balanced representation from most continents, expert groups and professions [22]. Recruitment occurred over an 8-week period (September 8 2021 to November 3 2021), with all interested individuals completing an expression of interest form, including demographic and expertise information [22]. Individuals who met the eligibility criteria were invited to participate.

**Table 1:** Participant eligibility criteria.

Expert Group	Inclusion Criteria
Researcher	≥2 peer reviewed publications relating to patient safety or adverse events and manipulation (SMT) and mobilization (MOB) in the previous 10 years
Manual therapy clinician	A clinical professional with ≥7 years of clinical practice experience using SMT/MOB to manage musculoskeletal conditions
Patient	An individual who has not received any training in SMT/MOB and has received SMT/MOB from a health care professional to manage a musculoskeletal condition
Medical doctor	A medical doctor who has a professional interest in SMT/MOB and/or adverse events following conservative treatments
Manual therapy student	A student actively enrolled in a professional program that includes SMT/MOB to manage musculoskeletal conditions
Professional regulatory body representative	An individual involved with local or federal policy and regulations for professions that use SMT/MOB to manage musculoskeletal conditions
Malpractice insurance representative	A professional malpractice insurance employee involved with malpractice claims for professions that use SMT/MOB to manage musculoskeletal conditions
Lawyer	A licensed legal professional with an interest in medico-legal actions involving adverse events following conservative treatment and/or professions that use SMT/MOB to manage musculoskeletal conditions
Data analyst or informatics/electronic health record representative	An individual with expertise in collecting standardized health data including, but not limited to adverse events, for professions that use SMT/MOB to manage musculoskeletal conditions

#### Focus groups.

All participants who responded to round 3 of the e-Delphi were invited to participate in a focus group following the conclusion of the e-Delphi process. Purposive sampling based on participant demographic characteristics, profession, geographical location, expert group and availability was used to ensure comparable exploration of findings from the e-Delphi process. This ensured diverse representation while still having enough participants in each focus group (minimum of 4 participants). All focus group participants signed a separate informed consent form and provided verbal consent for their focus group to be audio and video recorded.

### Protocol

The protocol for the e-Delphi process was registered at Open Science Framework (osf.io/ex3ha) in September 2021, and published *a priori* [22].

Due to the large heterogeneity of terms, definitions and classifications derived from round 1 responses, specific protocol deviations were deemed appropriate for the round 2 questionnaire:

- Limited feedback from round 1 responses was provided in round 2 for time efficiency (i.e., feedback was restricted to a) number of respondents and studies (from scoping review of the literature) that contributed to the development of questions included in round 2 and b) specification that terms included in this round’s questions derived from participant responses from round 1);
- Questions regarding participants’ preferences of terms were included based on terms that participants appeared to use interchangeably in round 1 responses; and
- Open text boxes were provided at the end of questionnaire sections (rather than after each statement) to allow participants to elaborate only when necessary (e.g., to clarify reasoning, suggest refinements, or raise concerns), thereby reducing cognitive burden.

Additional protocol deviations included:

- NH acted as the third reviewer for all qualitative analyses;
- Definitions from the International Classification of Functioning, Disability and Health (ICF) [25] and Serious Adverse Events (SAE) [26,27] were incorporated into round 2 and 3 questionnaires;
- Inferential statistics were not used to calculate stability in rounds 2 and 3 due to heterogeneity of responses; and

Focus groups were added to further understand and explain the results of the e-Delphi process.

## Data analysis

### e-Delphi process

Qualitative data from round 1 open-ended questions were analysed using a theoretical thematic analysis, using Braun and Clarke’s six-phase framework [28]. Initial coding was structured around domains pre-identified from the scoping review of the literature [19] and served as a deductive framework. Within each domain, data were examined inductively for any new themes [28,29]. Data were analysed independently by two researchers (MF/LG) using Microsoft Excel (Microsoft Corporation, USA) and any disagreements were resolved by discussion with a third reviewer (NH). The resulting codes were synthesized into statements for inclusion in Round 2 questionnaire. Wording used by participants was combined to generate statements that best represented similar themes across participants [30]. Statements generated from the scoping review [19] not identified from round 1 responses were included during round 2 questionnaire development. All researchers involved in round 1 open text responses are female and have a doctorate degree.

Both complete and incomplete responses were included in the analyses. Descriptive statistics, calculated using R (R Foundation for Statistical Computing, Vienna, Austria), were used to evaluate consensus in rounds 2 and 3 responses. Median, interquartile range and percentage agreement were calculated for each statement. Consensus for round 2 was pre-defined as 60% with an interquartile range (IQR) of ≤1.5 and for round 3, 70% with an IQR with of ≤1.0 [22,30–32], and represent the proportion of participants who rated a given statement as either “agree” or “strongly agree” on a 5-point Likert scale. Agreement of rounds 2 and 3 responses were assessed using Kendall’s coefficient of concordance to assess inter-rater agreement [22]. Round 3 responses were further sub-grouped by profession and expert group to evaluate sub-group consensus. Statements not achieving consensus in round 2 were not included in round 3. Open text comments provided by participants in rounds 2 and 3 were analyzed qualitatively using thematic analysis and were used to inform the focus group topic guide. Statements achieving consensus after round 3 were used to inform the final AE definition and severity classification reported in this manuscript.

### Focus groups

Data were analyzed once all focus groups had been completed using an inductive theoretical thematic analysis. The facilitator (MF) coded focus groups transcripts and identified key themes in consultation with a second reviewer (NH). Preliminary results were then presented to all named authors for further analysis and to inform interpretation. All researchers involved in focus groups data analysis are female, have a doctorate degree, and had previous experience with analysis of qualitative data.

## Results

### Participants

A total of 233 individuals expressed interest in participating in this study, with 215 meeting the eligibility criteria and being invited to participate in the e-Delphi process. Round 1 had 183 respondents (85% response rate), with 168 complete and 15 partially completed questionnaires. Subsequently, 212 participants were invited to round 2 with 163 (77%) responding (152 complete; 11 partial completion). Round 3 questionnaire was sent to 208 participants, of whom 153 (73%) responded (152 complete; 1 partial completion). Seven participants withdrew from the study (n = 5 due to availability of time; n = 2 due to methodological preferences/concerns).

At the end of round 3, a total of 74 participants (48%) expressed interest in focus group participation with a total of 16 participating.

Demographic characteristics of participants in each e-Delphi round and the focus groups are presented in Table 2. While all e-Delphi rounds had respondent representatives from all expert groups and most continents of the world, focus groups had representatives from most expert groups and continents.

**Table 2:** Participant demographic characteristics.

Characteristic	e-Delphi			Focus groups (n = 16)
	*Round 1 (n = 183)*	*Round 2 (n = 163)*	*Round 3 (n = 153)*	
**Age** (mean, min-max)	51 (24-80)	52 (25-80)	51 (30-80)	52 (31-69)
**Sex (n, %)**				
*Female*	61 (33.3)	56 (34.3)	50 (32.6)	6 (37.5)
**Manual Therapy profession (n, %)**				
*Chiropractic*	67 (36.6)	60 (36.8)	60 (39.2)	7 (43.5)
*Physiotherapy*	79 (43.1)	74 (45.4)	71 (46.4)	7 (43.5)
*Osteopathy*	8 (4.3)	12 (7.3)	9 (5.8)	1 (6.2)
*Naprapathy*	2 (1.1)	4 (2.4)	4 (2.5)	0
*Naturopathy*	1 (0.5)	1 (0.6)	1 (0.6)	0
**Continent (n, %)**				
*Africa*	4 (2.1)	4 (2.4)	4 (2.6)	1 (6.2)
*America*	122 (66.6)	115 (70.5)	107 (69.9)	10 (62.5)
*North America*	118	111	104	10
*South America*	4	4	3	0
*Asia*	1 (0.5)	1 (0.6)	2 (1.3)	0
*Europe*	37 (20.2)	30 (18.4)	26 (16.9)	3 (18.7)
*Oceania*	15 (8.2)	13 (7.9)	10 (6.5)	2 (12.5)
**Expert Group (n, %)**				
*Administration*	4 (2.1)	5 (3.0)	5 (3.2)	1 (6.2)
*Clinical Practice*	77 (42.0)	66 (40.5)	60 (39.2)	3 (18.7)
*Education/ Training*	46 (25.1)	45 (27.6)	46 (30.0)	9 (56.2)
*Malpractice Insurance*	3 (1.6)	3 (1.8)	3 (1.9)	1 (6.2)
*Patient*	4 (2.1)	3 (1.8)	1 (0.6)	0
*Practicing Law*	1 (0.5)	1 (0.6)	1 (0.6)	0
*Regulatory Body*	2 (1.1)	2 (1.2)	2 (1.3)	1 (6.2)
*Research*	36 (19.6)	31 (19.0)	28 (18.3)	1 (6.2)
*Student*	3 (1.6)	2 (1.2)	2 (1.3)	0
*Other*	33 (18.0)	3 (1.8)	3 (1.9)	0

### 1) e-Delphi process

#### Round 1.

##### Adverse event definition:

Common descriptors participants used to define AE following MAN and MOB included “unfavourable”, “unexpected”, “undesired”, and “unintended”. Domains commonly used to define AE were *temporality* (e.g., “during” or “after” treatment), *causality* (e.g., “result of the treatment” or “not necessarily caused by the treatment”) and *association* (e.g., “associated with the treatment”).

##### Adverse event severity classification:

Common categories participants used to classify the severity of AE following MAN and MOB included “benign”, “mild”, “minor”, “moderate”, “major”, “severe”, “serious”, “catastrophic” and “fatal”. Domains commonly used to describe differences between severity classification categories included *symptom intensity* (e.g., “low intensity” or “NRS score of 4-6”), *duration* (e.g., “lasting minutes to days” or “medium to long term”), *reversibility* (e.g., “reversible” or “permanent damage”), *level of disability* (e.g., “impairs function” or “incapacity”), *impact on patient* (e.g., “not interfering with normal activities” or “prohibits normal everyday life, work, and quality of life”) and *additional care required* (e.g., “requires medical attention” or “requiring medical interventions which could include surgery and hospitalization”).

#### Round 2.

##### Adverse event definition:

Ten terms reached consensus as most preferred by participants to define AE (i.e., “unfavourable”, “unanticipated”, “unexpected”, “undesired”, “unwanted”, “unintended”, “outcome”, “response”, “exacerbation”, and “following”). Similarly, 6 terms reached consensus as least preferred by participants (i.e., “deleterious”, “unusual”, “uncalled for”, “untoward”, “reaction”, and “deterioration”) and were discarded. Additionally, there was consensus to keep 5 statements and remove 3 statements (Table 3).

**Table 3:** Median, interquartile range (IQR), percentage agreement and Kendall’s coefficient of concordance for definition statements [1 = strongly agree/ 5 = strongly disagree].

Statement	Median	IQR	Percentage	Kendall’s coefficient of concordance
An adverse event is unfavourable.	1.7	1.0	85.8*	p < 0.00
An adverse event is expected.	4.0	2.0	13.0	
An adverse event is unexpected.	2.0	1.0	61.7*	
An adverse event is unintended.	1.0	1.0	84.0*	
An adverse event is undesired.	1.0	1.0	89.5*	
The development of new signs, symptoms or disease is an adverse event.	3.0	2.0	42.0	
The increase (or worsening) of signs, symptoms or disease is an adverse event.	2.0	1.0	56.8	
The persistence of signs, symptoms or disease is an adverse event.	4.0	2.0	11.1	
An adverse event can occur when no manipulation and/or mobilization is delivered (i.e., following assessments and/or examinations only).	2.0	1.0	78.4*	
An adverse event is unequivocally caused by spinal and/or peripheral joint manipulation and/or mobilization.	4.0	2.0	9.9	
“Adverse events related to manipulation and/or mobilization” has a similar meaning to “Adverse events caused by manipulation and/or mobilization”.	4.0	2.0	30.2	
“Adverse events associated with manipulation and/or mobilization” has similar meaning to “Adverse events caused by manipulation and/or mobilization”.	4.0	3.0	34.0	

*- Consensus reached (≥60.0% agreement).

##### Adverse event classification:

There was consensus that the terms “mild” and “minor” are similar (86%), with “mild” being the preferred term (79%). There was also consensus that the terms “major”, “severe” and “serious” are similar (72%), with “severe” being the preferred term (82%) and “catastrophic” having greater severity than “major”, “severe” and “serious” (93%). Additionally, there was consensus that *symptom intensity* (60%) and *impact on patient* (70%) were the most preferred domains to differentiate between severity categories. There was also consensus that *symptom duration* (48%), *symptom reversibility* (48%) and *additional care required* (49%) were the least preferred domains to differentiate between severity categories (Table 4). The domains that reached consensus to differentiate between AE severity categories are presented in Table 5. The descriptors suggested by most participants for each domain are also presented in Table 5 although consensus on the descriptors was not reached.

**Table 4:** Median, interquartile range (IQR), percentage agreement and Kendall’s coefficient of concordance for severity classification differentiator domains [1 = strongly agree/ 5 = strongly disagree].

Domain	Median	IQR	Percentage	Kendall’s coefficient of concordance
Intensity	2.0	1.0	60.0*	p < 0.00
Duration	3.0	2.0	48.1	
Reversibility	3.0	3.0	48.1	
Impact on a patient	2.0	1.0	70.0*	
Additional care required	3.0	2.0	48.7	

*- Consensus reached (≥60.0% agreement).

**Table 5:** Domains and their respective description to differentiate between severity classification categories.

Category	Symptom intensity		Impact on a patient	
	Words	Generic numeric 11-point scale	Words^	Generic scale^
**Mild**	Low	1-3	No impact Quality of life/participation	Tolerable – Some interference
**Moderate**	Medium	4-6	Quality of life/participation Activities/disability	Some interference – Considerable interference
**Severe**	High/ Important	7-10	Activities/disability	Considerable interference
**Catastrophic**	Significant	9	Life threatening Death	Total disruption

^ - Modified from ICF qualifier scale.

#### Round 3.

##### Adverse event definition:

None of the proposed statements related to the definition of AE following MAN and MOB reached consensus agreement (Table 6). Sub-grouped responses by profession and expert group also did not reach consensus [S4 Table].

**Table 6:** Frequency and percentage of most and least preferred; and median and interquartile range [IQR] of agreement [1 = strongly agree/ 5 = strongly disagree] for statements related to the definition and severity classification.

Definition		Most Preferred n (%)	Least Preferred n (%)	Agreement Median [IQR]
**An adverse event is any *unfavourable* outcome that occurs during or following spinal and/or peripheral manipulation and/or mobilization.**		79 (51.9)	53 (34.8)	1.0 [2.0]
**An adverse event is any *unfavourable and unexpected* outcome that occurs during or following spinal and/or peripheral manipulation and/or mobilization.**		26 (17.1)	33 (21.7)	2.0 [0.0]
**An adverse event is any *unfavourable, unexpected and undesired* outcome that occurs during or following spinal and/or peripheral manipulation and/or mobilization.**		48 (31.5)	67 (44.0)	2.0 [2.0]
**Severity Classification**		**Strongly Agree/ Agree n (%)**	**Disagree/ Strongly Disagree n (%)**	**Median [IQR]**
**“Benign” should not be used as an adverse event severity category as it relates to an outcome that is “not adverse”**		118 (77.6)*	20 (13.1)	2.0 [1.0]
*Domain*	*Description*			
**Symptom Intensity**	**A “mild” adverse event has a low intensity, ranging between 1–3 on an 11-point numeric scale**	140 (92.1)*	7 (4.6)	2.0 [1.0]
	**A “moderate” adverse event has a moderate intensity, ranging between 3–6 on an 11-point numeric scale**	137 (90.1)*	9 (5.9)	2.0 [1.0]
	**A “severe” adverse event has a high intensity, ranging between 6–8 on an 11-point numeric scale**	125 (82.2)*	18 (11.8)	2.0 [1.0]
	**A “catastrophic” adverse event has a significant intensity, ranging between 8–10 on an 11-point numeric scale**	104 (69.0)	34 (22.3)	2.0 [2.0]
**Impact on the patient**	**A “mild” adverse event has no impact on a patient’s activities, but it has a tolerable interference on participation, and quality of life**	120 (78.9)*	19 (12.5)	2.0 [1.0]
	**A “moderate” adverse event has some interference with a patient’s activities, participation, and quality of life**	147 (96.7)*	2 (1.3)	2.0 [1.0]
	**A “severe” adverse event is not life threatening, but has considerable interference with a patient’s activities, participation, and quality of life**	142 (93.4)*	8 (5.2)	2.0 [1.0]
	**A “catastrophic” adverse event is life-threatening and could result in death; it totally disrupts a patient’s activities, participation, and quality of life**	136 (89.4)*	10 (6.5)	1.0 [1.0]

*- Consensus reached (≥70.0% agreement).

##### Adverse event classification

There was consensus that the magnitude of *symptom intensity* and *impact on patient* would allow for differentiation between severity categories (Table 6).

A comprehensive table providing a summary of the severity classification description that reached consensus is provided in S5 Table.

### 2) Focus groups

Focus group discussions centered on and explored items that did not reach consensus during the e-Delphi process. For the definition of AE following MAN and MOB, participants shared opinions and thoughts related to the terms “unfavourable”, “unexpected” and “undesired”, and what AE meant to them. Participants discussed the possibility of expanding the AE definition beyond MAN and MOB to include physical examination of the patient and other conservative interventions.

With respect to severity categories, most participants stated that the term “catastrophic” implied greater severity than the term “serious”. Participants were presented with the SAE definition from the wider healthcare literature: “any untoward medical occurrence that results in death, is life-threatening requires inpatient hospitalization or causes prolongation of existing hospitalization results in persistent or significant disability/incapacity, may have caused a congenital anomaly/birth defect, or requires intervention to prevent permanent impairment or damage”. They were also presented with the definition from “catastrophic AE” from the e-Delphi process: “significant intensity, ranging between 8-10 on an 11-point numeric scale; life-threatening and could result in death; it totally disrupts a patient’s activities, participation, and quality of life”. When presented with both definitions, most participants stated that the definitions should be reversed. Participants also shared concerns regarding the: i) use of a generic numeric scale to classify AE as similar scales are commonly used by individuals to describe pain (AE are not only restricted to pain); ii) overlapping numeric scores related to symptom severity; iii) multiple criteria that must be met to determine severity category (i.e., symptom severity score and impact on patient); and iv) importance of including *symptom duration* to differentiate between severity categories (e.g., transient, permanent, etc.). A representation of the main themes discussed during the focus groups is presented in S1 Fig.

## Discussion

This study aimed to establish an inter-professional and international standardized definition and severity classification for AE following spinal and peripheral joint manipulation and mobilization for adults with musculoskeletal conditions and involved a large and diverse sample of participants representative of most expert stakeholder groups globally. Although consensus on a standardized definition for AE following MAN and MOB was not achieved, a standardized severity classification was established. Findings from focus groups emphasized the diversity of opinions contributing to the complexity of this topic.

### Adverse event definition

The current study builds on Carnes and colleagues’ (2010) Delphi process [33]with a larger, more diverse and representative sample in terms of professions that use MAN and MOB, geographical location and expert stakeholder groups. Interestingly, both studies did not reach consensus on a definition for AE. Collectively, these studies along with the previous scoping review reporting a vast array of AE definitions used throughout the literature [19] highlight the historical diversity and heterogeneity in defining AE following MAN and MOB. Indeed, the complexity of standardizing AE vocabulary has been described with some AE definitions evolving over time and being revised with knowledge advancements [34].

The challenge of establishing a standardized AE definition is not unique to MAN/MOB. Even main health-related resources have different definitions for AE. Specifically, the Common Terminology Criteria for Adverse Events (CTCAE) defines AE as an “unfavorable and unintended sign (including an abnormal laboratory finding), symptom, or disease temporally associated with the use of a medical treatment or procedure that may or may not be considered related to the medical treatment or procedure” [35]. Cochrane defines AE as an “unfavourable or harmful outcome that occurs during, or after, the use of a drug or other intervention, but is not necessarily caused by it” [36]. The World Health Organization (WHO) has more than 10 different definitions for AE in their conceptual framework for the international classification for patient safety document, including “an undesired patient outcome that may or may not be the result of an error” and “an injury that was caused by medical management and that results in measurable disability” [37]. It is noteworthy that most definitions include terms identified in our e-Delphi process (i.e., “unfavourable”, “unintended”, “undesired”); however, there were divergent opinions related to these terms in rounds 2 and 3, which were re-emphasized in the focus group discussions. While this could reflect individual preferences and different sources of AE knowledge and information, this lack of standardization is concerning as it can significantly influence the dialogue between healthcare professions and negatively impact patients’ safety and quality of care.

Focus group findings highlighted additional challenges related to language and heterogeneity in safety cultures depending on geographical location. This suggests that the diversity of opinions regarding the AE definition is influenced by local culture and standards. This is consistent with previous literature reporting on the influence of culture on health [38,39], as well as the WHO’s Global Patient Safety Action Plan Guiding Principles highlighting the importance of considering local context, culture, traditions, infrastructure and healthcare systems when implementing strategies to increase patient safety [10].

Focus group discussions revealed that some participants felt challenged with defining AE separately from a severity classification. This could be due to the close interconnection between defining AE based on their severity classification observed in previous studies [33,40–42]. Focus group discussions inferred that consensus would not be reached even if additional e-Delphi rounds were conducted. Discussions also provided participants with the opportunity to change their personal AE definition based other participants’ perspectives. This suggests that additional opportunities for discussions related to AE might assist with developing individual understandings and conceptualization. This, in turn, has the potential to contribute to establishing a standardized inter-professional and international definition for AE in the future and highlights the need for training institutions to implement these established definitions throughout their curriculum.

### Adverse event severity classification

The severity classification for AE following MAN and MOB and the domains used to differentiate categories that reached consensus in the current study are not aligned with those obtained in previous Delphi studies [33,43]. Specifically, one previous severity classification included “minor”, “moderate” and “major” categories with duration, severity and descriptor as domains [33]. Another severity classification included “no adverse event”, “minor” and “major” severity categories with duration as the domain [43]. While the differences could be explained by the participation of different respondents, the vast heterogeneity in AE classifications described in the literature likely also contributed. Given the wider professional, expert stakeholder group and global representation included in this study, the standardized severity classification for AE following MAN and MOB obtained from this e-Delphi constitutes a robust foundation for supporting a more standardized classification of AEs. This would facilitate AE reporting and dialogue that promote inter-professional learning significantly contributing to advancing patient safety.

Round 1 responses demonstrated the need to quantify the impact of AE on patients’ functionality, quality of life and well-being as domains to differentiate severity categories. As such, this study used ICF’s definitions and modified qualifier scale [25]. While this may facilitate the use of such a scale for MAN/MOB providers, additional studies are needed to further assess its applicability in the context of ICF standards.

Round 2 responses showed that participants deemed the terms “major”, “severe” and “serious” as similar. When the established SAE definition was incorporated in the round 3 questionnaire, it was not considered similar to “catastrophic”. Focus group discussions revealed that participants thought the term “catastrophic” implied greater severity than the term “serious” and some suggestions to address this issue were voiced, including having SAE as a separate definition altogether, as well as merging “severe” and “serious” categories or “catastrophic” and SAE. Given the importance of standardizing terminology across healthcare professions, further investigation into how SAE fit into the proposed severity classification is warranted.

Despite the domains used to differentiate between severity categories reaching consensus during the e-Delphi process, focus groups revealed potential concerns including: a) the 11-point generic numeric scale, b) the overlapping scores, and c) the importance of symptom duration (excluded in round 2).

a) The description of symptom intensity/severity using both words (e.g., “low”, “mild”, “moderate”, “high”, “significant”) and a generic 11-point numeric rating scale (NRS) have been observed not only in previous studies [33,41,44–47], but also during round 1 of this e-Delphi. The NRS is well-known to be a valid and reliable measurement of pain intensity and have been widely used in clinical practice and research in many healthcare areas [48–50]. Consequently, the assumption that the NRS included in this study’s severity classification was associated to pain intensity generated confusion in some participants. While the most common use of NRS is indeed for measuring pain intensity, especially in musculoskeletal conditions, it has also been validated for measuring other symptoms, such as perceived effort [51], spasticity [52], and itchiness [53]. In accordance with previous studies [44,45,54,55] and based on responses from round 1, the proposed NRS included in the severity classification of the current study was intended as a measure of overall symptom intensity, rather than pain specifically.
b) Open text comments throughout the e-Delphi process included the importance of considering patients’ perspectives. The subjectivity of symptom severity and the different impact of similar symptoms on different patients’ functionality and well-being has been described [56]. The overlapping numeric scores in our proposed severity classification could provide the flexibility to account for this subjectivity (S5 Table).
c) Given that *symptom duration* was incorporated as a domain to differentiate between severity categories in previous studies [33,40,43,44,46,57], it was interesting that this domain did not reach consensus in round 2 of the current study. While this could be associated with different severity classifications described in the literature that do not use *symptom duration* as a domain [45,58–60], focus group participants shared how important they believed it was to consider *symptom duration* when differentiating between severity categories. This highlights, once again, the unique individual opinions and perspectives that exist related to this topic.

While revisions should continue to be made as the here-established severity classification is validated, this study introduces a severity classification for AE following MAN and MOB that reached consensus among over 150 participants representing most continents, professions that use MAN and MOB and expert stakeholder groups.

### Strengths and limitations

Key strengths of this study included: i) adherence to an *a priori* published protocol [22]; ii) an inter-professional and international expert investigative team with patient representation; iii) the use of a sequential mixed-methods design that enabled further exploration of items that did not achieve consensus; iv) representation of all expert stakeholder groups and from most continents; and v) a high response rate and low attrition across all e-Delphi rounds. Limitations included: i) under representation from some expert groups (e.g., law, patients), manual therapy professions (e.g., naturopaths) and continents (e.g., Asia and Africa); ii) limited representation in the focus groups (n = 16) due to time, availability and organizational considerations; iii) although consensus was reached on a severity classification for AE following MAN and MOB, its validity and reliability remain unknown.

### Implications for practice and research

Findings contribute to advancing the knowledge related to AE following MAN and MOB internationally and across all professions that use these interventions. Even though a standardized adverse event (AE) definition was not reached, this study establishes a standardized severity classification for AEs following MAN and MOB internationally and across all professions that use these interventions. Additional opportunities for inter-professional and international discussions will contribute to advancing and evolving individuals’ perspectives and opinions, potentially towards a standardized AE definition in the future. Combined with the inter-professional and international standardized AE severity classification established in this study, such discussions will significantly contribute to enhancing precision in practice significantly enhancing patient safety and quality of care in clinical practice. Although future studies are needed to further assess the validity, reliability and applicability of the here-established severity classification and the incorporation of SAE definition, the classification developed in this study can serve as a preliminary reference to support more consistent inter-professional and international communication regarding AEs among all stakeholders.

## Conclusion

A standardized severity classification including mild, moderate, severe and catastrophic categories was established with detailed descriptors for each severity category. Consensus on a standardized definition for AE following MAN and MOB was not reached. Findings contribute to advancing patient safety and AE knowledge inter-professionally and internationally and inform further safety research to enhance patient safety in clinical practice.

## Supporting information

S1 Table Recommendations for the Conducting and REporting of DElphi Studies (CREDES). (DOCX)

S2 Table Standards for Reporting Qualitative Research (SRQR). (DOCX)

S3 Table Focus Group Topic Guide. (DOCX)

S4 Table Round 3 sub-group analysis. (DOCX)

S5 Table Comprehensive summary of established classifications that reached consensus in this study. (DOCX)

S1 File E-Delphi Questionnaires. (PDF)

S2 File Summary of e-Delphi results and discussion topics. (DOCX)

S1 Fig Representation of the main themes discussed during the focus groups. (DOCX)
