# Supplementary material for: Defining and classifying adverse events following joint manipulation and mobilization: An international e-Delphi study and focus groups
Source: PLoS One. 2025 Nov 17;20(11):e0334151. doi: 10.1371/journal.pone.0334151 (PMC12622795; doi:10.1371/journal.pone.0334151)
Supplement: S3 Table — (DOCX) [file pone.0334151.s003.docx]

**S3 Table**

**FOCUS GROUP – TOPIC GUIDE**

| **Focus group Section** | **Questions/Content** | **Prompts** |
| --- | --- | --- |
| **Ethics Statement** | Thank you for consenting to join this focus group. I am Martha and will facilitate the FG and with me is Nicola who is present as a moderator, and will be taking notes, keeping track of time, and monitoring the chat. Can I confirm that you all have read and understood the information sheet?  I’ll start with some instructions and ground rules:  This focus group will last about 90-120 minutes and will be audio and video-recorded (the chat will also be recorded). Please have your camera and microphone on at all times. Feel free to mute yourself in case you have background noise, but please unmute yourself as soon as the noise is gone. To avoid distractions, please make sure you are in a quiet environment, that you have your phone on silent and any other programs or applications on your computer (e.g., emails) closed. If you drop from the call, please connect again as soon as possible. All information shared today will be kept strictly confidential and I’ll ask you to please not share any information you hear here today with anyone.  You have the right to not answer a question if you do not wish to, but we will give everyone an opportunity to voice their opinions and we would like to hear everyone’s opinions. If, at any time, you are unsure about what is being asked or discussed, please let us know.  So, the aim of today’s focus group is to explore your thoughts, perceptions and opinions on the items that did not reach consensus at the end of round 3 of our Adverse Event Definition Delphi. So we are **not** aiming to reach consensus here, but instead better understanding **why** these did not reach consensus. Therefore, there are no right or wrong answers and all thoughts and opinions are welcome.  Before we start do you have any questions? |  |
| **Introductory Questions** | 1. Let’s start with some introductions… we’ll go around and if you could state your name, background, where do you work (academic, clinical practice, etc), your location?    - [facilitator and moderator introduce]    - Keep it succinct: name, research background, country      1. [“name, background and location” to be sent in the chat] |  |
| **Adverse event Delphi background** | As you know, this Delphi study aimed to determine, by an expert consensus process, a standardized definition and severity classification for “adverse events” following spinal and peripheral joint manipulation and mobilization. There were 3 rounds where the 1^st^ round consisted entirely of open-ended questions. The 2^nd^ round was developed based on the qualitative analysis of round 1 responses and a scoping review of the relevant literature and the 3^rd^ round narrowed the definition and classification based on round 2 responses.  At the end of round 3, there were two important items that did not reach consensus:   - the **definition and the classification** for “adverse events” following spinal and peripheral joint manipulation and mobilization |  |
| **Main Questions** | 1. Let’s start with the definition. The definitions provided in round 3 of the Delphi were the ones shown in the slide I am going to share with you right now:   [show slide with all 3 definitions]  I ask you to please pay attention to the highlighted words that are different in each of the definitions.  What are your thoughts about these words?  [PAUSE – give them time to think] | - *What do you like/agree with or don’t like/don't agree with about these definitions?* - *Do you think there is anything missing from these definitions?* - *Do you think there is something that should be removed from these definitions?* - *Did your thoughts related to adverse events definitions and classifications change throughout the study?* - *Did the feedback related to other participants’ responses influence your reflection on how you define adverse events?* - *In your opinion, are there any concerns related to how adverse events are defined?* |
|  | [Summarize or not the discussion on the definition – depending on how it goes]  Now let’s change topics:   1. What do you think about the terms or the words “serious” and “catastrophic” to be used as categories to classify the severity of adverse events?   [PAUSE – give time to think] | - *In your opinion, are there any differences between these terms?*   - *If yes, what are these differences?* - *What do you like/agree with or don’t like/don't agree with about these terms?* |
|  | 1. Do you feel there are any issues with using the terms “serious” versus “catastrophic” for classifying adverse events?   [PAUSE – give time to think] | - *Are there any limitations in using “serious”?* - *Are there any limitations in using “catastrophic”?* |
|  | 1. As we mentioned in the round 3 questionnaire, a "serious adverse event" (SAE) has an established definition within the wider healthcare field, as shown in this slide   [share slide with SAE definition]  Do you think this definition should be used for spinal and peripheral joint manipulation and mobilization?  [PAUSE – give time to think]   1. At the end of round 3, “catastrophic” AE had the definition shown in this slide   [share slide with SAE definition]  What do you think about this definition in comparison to the SAE? | - *What do you like/agree with or don’t like/don't agree with about this definition?* - *Do you think there is anything missing in this definition?* - *Do you think there is something that should be removed from this definition?* - *Do you think there are any limitations with using this “serious adverse event” definition?* |
|  | 1. What do you think influences your thoughts and opinions related to the adverse event definition? | - *Do you think personal and professional background influences how you define adverse events?* - *Do you think your experience in your field/area influences how you define adverse events?* |
| **Conclusion** | That’s all the questions, is there anything else you would like to add regarding the adverse event definition and/or the use of “serious” versus “catastrophic” as categories?  The focus group has now finished. We will be conducting other focus groups and will combine the feedback you have provided with that gathered in the other groups. Thank you for participating in this study, we really appreciate your time and input. |  |
