# Supplementary material for: Defining and classifying adverse events following joint manipulation and mobilization: An international e-Delphi study and focus groups
Source: PLoS One. 2025 Nov 17;20(11):e0334151. doi: 10.1371/journal.pone.0334151 (PMC12622795; doi:10.1371/journal.pone.0334151)
Supplement: S4 Table — (DOCX) [file pone.0334151.s004.docx]

**S4 Tables**

**Round 3 sub-group analysis**

**By Profession**

Please rank the following statements indicating your preferred **definition** for adverse event:

| **Definition** | **Physiotherapy (n=71)** | | **Chiropractic (n=63)** | | **Osteopathy (n=9)** | | **Naprapathy (n=4)** | | **Medicine (n=4)** | |
| --- | --- | --- | --- | --- | --- | --- | --- | --- | --- | --- |
|  | **Most Preferred** | **Least Preferred** | **Most Preferred** | **Least Preferred** | **Most Preferred** | **Least Preferred** | **Most Preferred** | **Least Preferred** | **Most Preferred** | **Least Preferred** |
| An adverse event is any unfavourable outcome that occurs during or following spinal and/or peripheral manipulation and/or mobilization. | 32 (45.1%) | 30 (42.2%) | 36 (57.1%) | 18 (28.5%) | 4  (44.4%) | 3  (33.3%) | 3  (75.0%) | 1  (25.0%) | 3  (75.0%) | 0 |
| An adverse event is any unfavourable and unexpected outcome that occurs during or following spinal and/or peripheral manipulation and/or mobilization. | 12 (16.9%) | 8  (11.2%) | 11 (17.4%) | 16 (25.4%) | 2  (22.2%) | 4  (44.4%) | 0 | 1  (25.0%) | 1  (25.0%) | 3  (75.0%) |
| An adverse event is any unfavourable, unexpected and undesired outcome that occurs during or following spinal and/or peripheral manipulation and/or mobilization. | 27 (38.0%) | 33 (46.5%) | 16 (25.4%) | 29 (46.0%) | 3  (33.3%) | 2  (22.2%) | 1  (25.0%) | 2  (50.0%) | 0 | 1  (25.0%) |

For the next questions, please indicate your level of agreement with the following statements, regardless of your level of agreement with the previous statements.

| **Statement** | **Physiotherapy (n=71)** | **Chiropractic (n=63)** | **Osteopathy (n=9)** | **Naprapathy (n=4)** | **Medicine (n=4)** |
| --- | --- | --- | --- | --- | --- |
|  | **Strongly Agree / Agree** | **Strongly Agree / Agree** | **Strongly Agree / Agree** | **Strongly Agree / Agree** | **Strongly Agree / Agree** |
| A “catastrophic” adverse event has a significant intensity, ranging between 8-10 on an 11-point numeric scale | 49  (69.0%) | 42  (66.6%) | 8  (88.8%) | 3  (75.0%) | 1  (25.0%) |

Serious and Catastrophic Adverse Events

| **Statement** | **Physiotherapy (n=71)** | **Chiropractic (n=63)** | **Osteopathy (n=9)** | **Naprapathy (n=4)** | **Medicine (n=4)** |
| --- | --- | --- | --- | --- | --- |
|  | **Strongly Agree / Agree** | **Strongly Agree / Agree** | **Strongly Agree / Agree** | **Strongly Agree / Agree** | **Strongly Agree / Agree** |
| A "serious adverse event" (SAE) has an established definition within the wider healthcare field, that is:  "Any untoward medical occurrence that results in death, is life-threatening requires inpatient hospitalization or causes prolongation of existing hospitalization results in persistent or significant disability/incapacity, may have caused a congenital anomaly/birth defect, or requires intervention to prevent permanent impairment or damage."[3-7]  At this time, data from this study suggest that a "catastrophic" adverse event "has significant intensity, ranging between 8-10 on an 11-point numeric scale; is life-threatening and could result in death; it totally disrupts a patient's activities, participation, and quality of life."  Please indicate your level of agreement that these two definitions should be considered similar. | 47  (66.2%) | 41  (65.1%) | 7  (77.7%) | 3  (75.0%) | 2  (50.0%) |

**By Expert Group**

Please rank the following statements indicating your preferred **definition** for adverse event:

| **Definition** | **Clinical Practice (n=60)** | | **Education / Training (n=46)** | | **Research (n=29)** | |
| --- | --- | --- | --- | --- | --- | --- |
|  | **Most Preferred** | **Least Preferred** | **Most Preferred** | **Least Preferred** | **Most Preferred** | **Least Preferred** |
| An adverse event is any unfavourable outcome that occurs during or following spinal and/or peripheral manipulation and/or mobilization. | 28 (46.6%) | 23 (38.3%) | 25  (54.3%) | 16  (34.8%) | 18 (62.0%) | 8  (27.6%) |
| An adverse event is any unfavourable and unexpected outcome that occurs during or following spinal and/or peripheral manipulation and/or mobilization. | 8  (13.3%) | 14 (23.3%) | 8  (17.4%) | 8  (17.4%) | 6  (20.7%) | 7  (24.1%) |
| An adverse event is any unfavourable, unexpected and undesired outcome that occurs during or following spinal and/or peripheral manipulation and/or mobilization. | 24 (40.0%) | 23 (38.3%) | 13  (28.2%) | 22  (47.8%) | 5  (17.2%) | 14 (48.3%) |

For the next questions, please indicate your level of agreement with the following statements, regardless of your level of agreement with the previous statements.

| **Statement** | **Clinical Practice (n=60)** | **Education / Training (n=46)** | **Research (n=29)** |
| --- | --- | --- | --- |
|  | **Strongly Agree / Agree** | **Strongly Agree / Agree** | **Strongly Agree / Agree** |
| A “catastrophic” adverse event has a significant intensity, ranging between 8-10 on an 11-point numeric scale | 44  (73.3%) | 33  (71.7%) | 14  (48.3%) |

Serious and Catastrophic Adverse Events

| **Statement** | **Clinical Practice (n=60)** | **Education / Training (n=46)** | **Research (n=29)** |
| --- | --- | --- | --- |
|  | **Strongly Agree / Agree** | **Strongly Agree / Agree** | **Strongly Agree / Agree** |
| A "serious adverse event" (SAE) has an established definition within the wider healthcare field, that is:  "Any untoward medical occurrence that results in death, is life-threatening requires inpatient hospitalization or causes prolongation of existing hospitalization results in persistent or significant disability/incapacity, may have caused a congenital anomaly/birth defect, or requires intervention to prevent permanent impairment or damage."[3-7]  At this time, data from this study suggest that a "catastrophic" adverse event "has significant intensity, ranging between 8-10 on an 11-point numeric scale; is life-threatening and could result in death; it totally disrupts a patient's activities, participation, and quality of life."  Please indicate your level of agreement that these two definitions should be considered similar. | 46  (76.6%) | 29  (63.0%) | 17  (58.6%) |
