# Supplementary material for: Defining and classifying adverse events following joint manipulation and mobilization: An international e-Delphi study and focus groups
Source: PLoS One. 2025 Nov 17;20(11):e0334151. doi: 10.1371/journal.pone.0334151 (PMC12622795; doi:10.1371/journal.pone.0334151)
Supplement: S5 Table — (DOCX) [file pone.0334151.s005.docx]

**S5 Table**

**Comprehensive summary of established severity classifications that reached consensus in this study.**

| Category | Intensity | Impact on patient |
| --- | --- | --- |
| MILD | Low intensity, ranging between 1-3 on an 11-point numeric scale. | No impact on a patient’s activities, but has a tolerable interference on participation, and quality of life. |
| MODERATE | Moderate intensity, ranging between 3-6 on an 11-point numeric scale. | Some interference with a patient’s activities, participation, and quality of life. |
| SEVERE | High intensity, ranging between 6-8 on an 11-point numeric scale. | Not life threatening, but has considerable interference with a patient’s activities, participation, and quality of life. |
| CATASTROPHIC | Significant intensity, ranging between 8-10 on an 11-point numeric scale. | Life-threatening and could result in death, totally disrupts a patient’s activities, participation, and quality of life. |

Note: The overlapping numeric scores provides the flexibility to account for symptom subjectivity. For example, if a patient reported an adverse event with symptom intensity of 3 (overlapping between a “mild” and a “moderate” severity), the impact of the symptom on the patient’s activities, participation and quality of life could be used to determine the adverse event severity.
