# Supplementary material for: Defining and classifying adverse events following joint manipulation and mobilization: An international e-Delphi study and focus groups
Source: PLoS One. 2025 Nov 17;20(11):e0334151. doi: 10.1371/journal.pone.0334151 (PMC12622795; doi:10.1371/journal.pone.0334151)
Supplement: S1 File — (PDF) [file pone.0334151.s006.pdf]

S1 File
e-Delphi Questionnaires

Note: questionnaires included branching logic and skip patterns and some questions appeared (or not) depending on respondents' previous answers.

Round 1

Please complete the questionnaire below.

Thank you!

Expert Consensus on a Standardized Definition and Classification for Adverse Events Associated with Spinal and Peripheral Joint Manipulation and Mobilization: An International E-Delphi Study

ROUND 1

Spinal and peripheral joint manipulation and mobilization are interventions commonly used by a variety of healthcare professionals (e.g. chiropractors, naprapaths, naturopaths, osteopaths, physiotherapists, etc.) in the management of many musculoskeletal conditions, including spinal pain, and are most often administered in ambulatory care settings. These interventions are described by a vast array of terms, including high-velocity low-amplitude manipulation, low-velocity variable-amplitude mobilization, spinal manipulative therapy, musculoskeletal manipulation/mobilization, etc.

In this study:

[Technical definitions]
Manipulation refers to a passive, high velocity low amplitude thrust applied to a joint complex within its anatomical limit with the intent to restore optimal motion, function, and/or to reduce pain; and

Mobilization refers to a passive articulatory movement applied to a single joint or joints in close proximity to eachother with the intent to restore optimal motion, function, and/or to reduce pain [1].

[Lay definitions]
Manipulation (also called adjustment) refers to a treatment that involves the therapist applying one rapid force/thrust to a joint with the aim of relieving pain or stiffness; and

Mobilization refers to a treatment that involves the therapist applying a force repeatedly (at variable speeds and amplitudes) to a joint with the aim of relieving pain or stiffness.

Spinal refers to joints of the neck, mid and low back.

Peripheral joint refers to shoulder, arms, hands, fingers, hip, leg, foot and toes.

If, at any time during this questionnaire, you are unsure about any definition, please feel free to contact Martha Funabashi at mfunabashi@cmcc.ca.

1- Rushton A, Beeton K, Jordaan R, Langendoen J, Levesque L, Maffey L, Pool J (2016). IFOMPT Educational Standards Document. IFOMPT.

Part 1A - Adverse Event Definition

How do you define an "adverse event"?

____

What other terms or words do you use, that are similar to, or refer to an "adverse event"?

____

If you have any questions about any definition, please contact Martha Funabashi at mfunabashi@cmcc.ca.

Part 1A - Adverse Event Definition Cont.

Would you use a different "adverse event" definition for:

	Yes	No
"manipulation" versus "mobilization"?	◯	◯
"spinal" versus "peripheral" joint manipulation/mobilization	◯	◯

Please provide your definition for an "adverse event" following:
spinal or peripheral joint manipulation or mobilization?
(please give examples where possible) ________________________________

Please provide your definition for an "adverse event" following:
spinal manipulation or mobilization?
(please give examples where possible) ________________________________

Please provide your definition for an "adverse event" following:
peripheral joint manipulation or mobilization?
(please give examples where possible) ________________________________

Please provide your definition for an "adverse event" following:
spinal or peripheral joint manipulation?
(please give examples where possible) ________________________________

Please provide your definition for an "adverse event" following:
spinal or peripheral joint mobilization?
(please give examples where possible) ________________________________

Please provide your definition for an "adverse event" following:
spinal manipulation?
(please give examples where possible) ________________________________

Please provide your definition for an "adverse event" following:
peripheral joint manipulation?
(please give examples where possible) ________________________________

Please provide your definition for an "adverse event" following:
spinal mobilization?
(please give examples where possible) ________________________________

Please provide your definition for an "adverse event" following:
peripheral joint mobilization?
(please give examples where possible) ________________________________

Please provide references, citations, or resources from which your "adverse event" definitions were derived.
(e.g., references/citations, researcher/scientist/academic/organizations) ______________________________________

If you have any questions about any definition, please contact Martha Funabashi at mfunabashi@cmcc.ca.

Part 1B - Adverse Event Classification

How do you classify "adverse events"?

Please provide a description for each classification stated. ____

If you have any questions about any definition, please contact Martha Funabashi at mfunabashi@cmcc.ca.

Part 1C - Adverse Event Severity Classification

For the following questions, we are focusing on the severity classification of "adverse events":

Please select the word(s) you use to classify the severity of "adverse events" following spinal and peripheral joint manipulation and mobilization: (Select all that apply)

- ☐ Benign
- ☐ Catastrophic
- ☐ Fatal
- ☐ Major
- ☐ Mild
- ☐ Minor
- ☐ Moderate
- ☐ Serious
- ☐ Severe
- ☐ Other
- ☐ None

Please specify the "other" word(s): __________________________________

How would you define a benign adverse event? __________________________________

Please provide example(s) of a benign adverse event. __________________________________

How would you define a catastrophic adverse event? __________________________________

Please provide example(s) of a catastrophic adverse event. __________________________________

How would you define a fatal adverse event? __________________________________

Please provide example(s) of a fatal adverse event. __________________________________

How would you define a major adverse event? __________________________________

Please provide example(s) of a major adverse event. __________________________________

How would you define a mild adverse event? __________________________________

Please provide example(s) of a mild adverse event. __________________________________

How would you define a minor adverse event?

____

Please provide example(s) of a minor adverse event.

____

How would you define a moderate adverse event?

____

Please provide example(s) of a moderate adverse event.

____

How would you define a serious adverse event?

____

Please provide example(s) of a serious adverse event.

____

How would you define a severe adverse event?

____

Please provide example(s) of a severe adverse event.

____

How would you define a [sev_class_categ_oth] adverse event?

____

Please provide example(s) of a [sev_class_categ_oth] adverse event.

____

Please provide references, citations, or resources that support the responses you provided, if applicable.

____

If you have any questions about any definition, please contact Martha Funabashi at mfunabashi@cmcc.ca.

Part 2 - General Comments

Please provide any further comments you feel are relevant regarding ''adverse events'' definition and/or their classification. __________________________________

Thank you!

This is the end of the round 1 questionnaire. Thank you for your participation and contribution.

Round 1 is open until February 15th, 2022. We will analyse the results and develop a round 2 questionnaire using the statements you and other participants have provided, as well as results from a scoping review of the literature. We will provide you with a link to the round 2 questionnaire about 3 weeks after round 1 is complete.

Thank you for your time and commitment in assisting us to standardize the definition and severity classification of "adverse events" following spinal and peripheral joint manipulation and mobilization.

Best Regards,

Martha Funabashi, Lindsay Gorrell, Katie Pohlman, Andrea Bergna, Nicola Heneghan

If you have any questions, please contact: Martha Funabashi (mfunabashi@cmcc.ca)

AE Delphi - Round 2

EXPERT CONSENSUS ON A STANDARDIZED DEFINITION AND SEVERITY CLASSIFICATION FOR ADVERSE EVENTS FOLLOWING SPINAL AND PERIPHERAL JOINT MANIPULATION AND MOBILIZATION: AN INTERNATIONAL E-DELPHI STUDY

ROUND 2 Investigators:
- Martha Funabashi, BSc, MSc, PhD - Canadian Memorial Chiropractic College (Canada)
- Lindsay M Gorrell, BChiroSc, MChiroprac, MRes, PhD - University of Zurich (Switzerland)
- Katherine A Pohlman, DC, MS, PhD - Parker University (USA)
- Andrea Bergna, Bsc, PT, DO - SOMA Instituto Osteopatia Milano (Italy)
- Nicola R Heneghan, PhD, MSc, PG Cert HEd, Grad Dip Phys - University of Birmingham (England)

REMINDER of study terms:
Spinal and peripheral joint manipulation and mobilization are interventions commonly used by a variety of healthcare professionals (e.g. chiropractors, naprapaths, naturopaths, osteopaths, physiotherapists, etc.) in the management of many musculoskeletal conditions, including spinal pain, and are most often administered in ambulatory care settings. These interventions are described by a vast array of terms, including high-velocity low-amplitude manipulation, low-velocity variable-amplitude mobilization, spinal manipulative therapy, musculoskeletal manipulation/mobilization, etc.

In this study: Manipulation (also called adjustment) refers to a treatment that involves the therapist applying one rapid force/thrust to a joint with the aim of relieving pain or stiffness; and

Mobilization refers to a treatment that involves the therapist applying a force repeatedly (at variable speeds and amplitudes) to a joint with the aim of relieving pain or stiffness.

Spinal refers to joints of the neck, mid and low back.

Peripheral joint refers to shoulders, arms, hands, fingers, hips, legs, feet and toes.

If, at any time during this questionnaire, you have any questions, please feel free to contact Martha Funabashi at mfunabashi@cmcc.ca.

Round 2 questions were developed based on findings from round 1 data (180 respondents) and a scoping review of the literature (69 studies) related to the definition and severity classification for adverse events following spinal and/or peripheral joint manipulation and/or mobilization. The questions include terms that were used by respondents and in the literature to define and classify the severity of adverse events following spinal and/or peripheral joint manipulation and/or mobilization. Estimated time for completion: 40 minutes.

DEFINITION

The following questions relate to the definition for adverse events following spinal and/or peripheral joint manipulation and/or mobilization.

Please rank your level of preference for each of the terms below:

	1-Most preferred	2	3	4	5-Least preferred
Deleterious	○	○	○	○	○
Detrimental	○	○	○	○	○
Negative	○	○	○	○	○
Unfavourable	○	○	○	○	○

Unpleasant	○	○	○	○	○

Please rank your level of preference for each of the terms below:

	1-Most preferred	2	3	4-Least preferred
Unanticipated	◯	◯	◯	◯
Uncommon	◯	◯	◯	◯
Unexpected	◯	◯	◯	◯
Unusual	◯	◯	◯	◯

Please consider the following terms:

Which is your preferred term: ◯ Common ◯ Expected

Please rank your level of preference for each of the terms below:

	1-Most preferred	2	3	4-Least preferred
Uncalled for	○	○	○	○
Undesired	○	○	○	○
Untoward	○	○	○	○
Unwanted	○	○	○	○

Please rank your level of preference for each of the terms below:

	1-Most preferred	2	3-Least preferred
Unintended	○	○	○
Unplanned	○	○	○
Unwanted	○	○	○

Please rank your level of preference for each of the terms below:

	1-Most preferred	2	3	4-Least preferred
Effect	◯	◯	◯	◯
Outcome	◯	◯	◯	◯
Reaction	◯	◯	◯	◯
Response	◯	◯	◯	◯

Please rank your level of preference for each of the terms below:

	1-Most preferred	2	3	4	5-Least preferred
Aggravation	◯	◯	◯	◯	◯
Deterioration	◯	◯	◯	◯	◯
Exacerbation	◯	◯	◯	◯	◯
Increased	◯	◯	◯	◯	◯
Worsened	◯	◯	◯	◯	◯

Please consider the following terms:

Which is your preferred term: ◯ After ◯ Following

Please indicate how much you agree with the statements below:

	Strongly Agree	Agree	Neutral	Disagree	Strongly Disagree
An adverse event is unfavourable.	○	○	○	○	○
An adverse event is expected.	○	○	○	○	○
An adverse event is unexpected.	○	○	○	○	○
An adverse event is unintended.	○	○	○	○	○
An adverse event is undesired.	○	○	○	○	○
The development of new signs, symptoms or disease is an adverse event.	○	○	○	○	○
The increase (or worsening) of signs, symptoms or disease is an adverse event.	○	○	○	○	○
The persistence of signs, symptoms or disease is an adverse event.	○	○	○	○	○
An adverse event can occur when no manipulation and/or mobilization is delivered (i.e., following assessments and/or examinations only).	○	○	○	○	○

Please read and apply the following operational definitions for the questions below:

Temporality: The state of existing within or having some relationship with time. For example, an outcome (i.e., adverse event) occurs after an exposure (i.e., spinal and/or peripheral joint manipulation and/or mobilization).

Causality: The relationship between cause and effect. For example, an exposure (i.e., spinal and/or peripheral joint manipulation and/or mobilization) caused the outcome (i.e., adverse event). Scientifically, the preferred research methodology to investigate causal relationships between an exposure and outcome is the randomized controlled trial where the exposure of interest and its relationship to the outcome is measured.[1]
Please note that for causality to be unequivocally established, the following criteria should be used: strength of association, consistency, specificity, temporality, biologic gradient, plausibility, coherence, experimental evidence, and analogy.[2]

[1] Cartwright. What are randomised controlled trials good for? Philos Stud, 2010; 147:59-70.
[2] Rothman & Greenland. Hill's criteria for causality. Encyclopedia of Biostatistics, 2005.

Please indicate how much you agree with the statements below:

	Strongly Agree	Agree	Neutral	Disagree	Strongly Disagree
An adverse event has a temporal relationship with spinal and/or peripheral joint manipulation and/or mobilization.	◯	◯	◯	◯	◯
An adverse event is unequivocally caused by spinal and/or peripheral joint manipulation and/or mobilization.	◯	◯	◯	◯	◯
"Adverse events related to manipulation and/or mobilization" has a similar meaning to "Adverse events caused by manipulation and/or mobilization".	◯	◯	◯	◯	◯
"Adverse events associated with manipulation and/or mobilization" has similar meaning to "Adverse events caused by manipulation and/or mobilization".	◯	◯	◯	◯	◯

How is it possible to unequivocally determine if an adverse event was caused by spinal and/or peripheral joint manipulation and/or mobilization? ______________________________

Please indicate if you agree with the statements below:

	Yes	No
"Unfavourable" and "deleterious" are similar.	○	○
"Unfavourable" and "detrimental" are similar.	○	○
"Unfavourable" and "negative" are similar.	○	○
"Unfavourable" and "unpleasant" are similar.	○	○

Please indicate if you agree with the statements below:

	Yes	No
"Unexpected" and "unanticipated" are similar.	○	○
"Unexpected" and "uncommon" are similar.	○	○
"Unexpected" and "unusual" are similar.	○	○

Please indicate if you agree with the following statement:

"Expected" and "common" are similar. ◯ Yes ◯ No

Please indicate if you agree with the statements below:

	Yes	No
"Undesired" and "uncalled for" are similar.	◯	◯
"Undesired" and "untoward" are similar.	◯	◯
"Undesired" and "unwanted are similar.	◯	◯

Please indicate if you agree with the statements below:

	Yes	No
"Unintended" and "unplanned" are similar.	○	○
"Unintended" and "unwanted" are similar.	○	○

Please indicate if you agree with the statements below:

	Yes	No
"Outcome" and "effect" are similar.	○	○
"Outcome" and "reaction" are similar.	○	○
"Outcome" and "response" are similar.	○	○

Please indicate if you agree with the statements below:

	Yes	No
"Increased (sign, symptom or disease)" and "aggravation of (sign, symptom or disease)" are similar.	◯	◯
"Increased (sign, symptom or disease)" and "deterioration of (sign, symptom or disease)" are similar.	◯	◯
"Increased (sign, symptom or disease)" and "exacerbation of (sign, symptom or disease)" are similar.	◯	◯
"Increased (sign, symptom or disease)" and "worsened (sign, symptom or disease)" are similar.	◯	◯

Please indicate if you agree with the following statement:

"Following spinal and/or peripheral joint manipulation and/or mobilization" and "after spinal and/or peripheral joint manipulation and/or mobilization" are similar.

◯ Yes ◯ No

Thank you for your responses so far. The next section includes example definitions based on our earlier findings.

Please keep the following operational definitions in mind for the next section:

Temporality: The state of existing within or having some relationship with time. For example, an outcome (i.e., adverse event) occurs after an exposure (i.e., spinal and/or peripheral joint manipulation and/or mobilization).

Causality: The relationship between cause and effect. For example, an exposure (i.e., spinal and/or peripheral joint manipulation and/or mobilization) caused the outcome (i.e., adverse event).[1]
Please note that for causality to be unequivocally established, the following criteria should be used: strength of association, consistency, specificity, temporality, biologic gradient, plausibility, coherence, experimental evidence, and analogy.[2]

[1] Cartwright. What are randomised controlled trials good for? Philos Stud, 2010; 147:59-70.
[2] Rothman & Greenland. Hill's criteria for causality. Encyclopedia of Biostatistics, 2005

Please rate the following definitions:

	Agree as stated	Needs modification
"An adverse event is defined as any expected or unexpected, unfavourable, undesired and unintended outcome that occurs during or after spinal and/or peripheral joint manipulation and/or mobilization, whether or not caused by that manipulation and/or mobilization."	○	○
"An adverse event is defined as any new or increased sign, symptom or disease that occurs during or after spinal and/or peripheral joint manipulation and/or mobilization, whether or not caused by that manipulation and/or mobilization."	○	○

How would you change the proposed definition?

"An adverse event is defined as any expected or unexpected, unfavourable, undesired and unintended outcome that occurs during or after spinal and/or peripheral joint manipulation and/or mobilization, whether or not caused by that manipulation and/or mobilization."

____

How would you change the proposed definition?

"An adverse event is defined as any new or increased sign, symptom or disease that occurs during or after spinal and/or peripheral joint manipulation and/or mobilization, whether or not caused by that manipulation and/or mobilization."

____

Comment section

Please provide any additional thoughts or comments regarding the definition for adverse events following spinal and/or peripheral joint manipulation and/or mobilization. __________________________________

SEVERITY CLASSICATION

The following questions relate to the severity classification for adverse events following spinal and/or peripheral joint manipulation and/or mobilization.

Please indicate if you agree with the statements below:

	Yes	No
"Benign" and "mild" are similar.	◯	◯
"Benign" and "minor" are similar.	◯	◯
"Mild" and "minor" are similar.	◯	◯

Which is your preferred term?	◯ Benign<br>◯ Mild
Which one is your preferred term?	◯ Benign<br>◯ Minor
Which is your preferred term?	◯ Mild<br>◯ Minor
Which is your "least preferred" term?	◯ Benign<br>◯ Mild<br>◯ Minor
Which is your "most preferred" term?	◯ Benign<br>◯ Mild<br>◯ Minor
Which would you rate as the "least severe"?	◯ Benign / Mild<br>◯ Minor
Which would you rate as the "least severe"?	◯ Benign / Minor<br>◯ Mild
Which would you rate as the "least severe"?	◯ Benign<br>◯ Mild / Minor
Which would you rate as the "least severe"?	◯ Benign<br>◯ Mild<br>◯ Minor
Which would you rate as the "most severe"?	◯ Benign<br>◯ Mild<br>◯ Minor

Please indicate if you agree with the statements below:

	Yes	No
"Catastrophic" and "major" are similar.	◯	◯
"Catastrophic" and "severe" are similar.	◯	◯
"Catastrophic" and "serious" are similar.	◯	◯
"Major" and "severe" are similar.	◯	◯
"Major" and "serious" are similar.	◯	◯
"Severe" and "serious" are similar.	◯	◯

Which is your preferred term?
◯ Catastrophic
◯ Major

Which is your preferred term?
◯ Catastrophic
◯ Severe

Which is your preferred term?
◯ Catastrophic
◯ Serious

Which is your preferred term?
◯ Major
◯ Severe

Which is your preferred term?
◯ Major
◯ Serious

Which is your preferred term?
◯ Severe
◯ Serious

Which is your "least preferred" term?
◯ Catastrophic
◯ Major
◯ Severe

Which is your "most preferred" term?
◯ Catastrophic
◯ Major
◯ Severe

Which is your "least preferred" term?
◯ Catastrophic
◯ Major
◯ Serious

Which is your "most preferred" term?
◯ Catastrophic
◯ Major
◯ Serious

Which is your "least preferred" term?
◯ Catastrophic
◯ Severe
◯ Serious

Which is your "most preferred" term?	◯ Catastrophic<br>◯ Severe<br>◯ Serious
Which is your "least preferred" term?	◯ Major<br>◯ Severe<br>◯ Serious
Which is your "most preferred" term?	◯ Major<br>◯ Severe<br>◯ Serious
Which is your "least preferred" term?	◯ Catastrophic<br>◯ Major<br>◯ Severe<br>◯ Serious
Which is your "most preferred" term?	◯ Catastrophic<br>◯ Major<br>◯ Severe<br>◯ Serious
Which would you rate as the "least severe"?	◯ Catastrophic / Severe / Serious<br>◯ Major
Which would you rate as the "least severe"?	◯ Catastrophic / Major / Serious<br>◯ Severe
Which would you rate as the "least severe"?	◯ Catastrophic / Major / Severe<br>◯ Serious
Which would you rate as the "least severe"?	◯ Major / Severe / Serious<br>◯ Catastrophic
Which would you rate as the "least severe"?	◯ Catastrophic / Major<br>◯ Severe / Serious
Which would you rate as the "least severe"?	◯ Catastrophic / Severe<br>◯ Major / Serious
Which would you rate as the "least severe"?	◯ Catastrophic / Serious<br>◯ Major / Severe
Which would you rate as the "least severe"?	◯ Catastrophic / Major<br>◯ Severe<br>◯ Serious
Which would you rate as the "most severe"?	◯ Catastrophic / Major<br>◯ Severe<br>◯ Serious
Which would you rate as the "least severe"?	◯ Catastrophic / Severe<br>◯ Major<br>◯ Serious

Which would you rate as the "most severe"?	◯ Catastrophic / Severe<br>◯ Major<br>◯ Serious
Which would you rate as the "least severe"?	◯ Catastrophic / Serious<br>◯ Major<br>◯ Severe
Which would you rate as the "most severe"?	◯ Catastrophic / Serious<br>◯ Major<br>◯ Severe
Which would you rate as the "least severe"?	◯ Major / Severe<br>◯ Catastrophic<br>◯ Serious
Which would you rate as the "most severe"?	◯ Major / Severe<br>◯ Catastrophic<br>◯ Serious
Which would you rate as the "least severe"?	◯ Major / Serious<br>◯ Catastrophic<br>◯ Severe
Which would you rate as the "most severe"?	◯ Major / Serious<br>◯ Catastrophic<br>◯ Severe
Which would you rate as the "least severe"?	◯ Severe / Serious<br>◯ Catastrophic<br>◯ Major
Which would you rate as the "most severe"?	◯ Severe / Serious<br>◯ Catastrophic<br>◯ Major
Which would you rate as the "least severe"?	◯ Catastrophic<br>◯ Major<br>◯ Severe<br>◯ Serious
Which would you rate as the "most severe"?	◯ Catastrophic<br>◯ Major<br>◯ Severe<br>◯ Serious

Core messages

The next section includes several domains derived from earlier findings.

Please rate your level of preference for each domain that you think should be used to differentiate severity categories:

	1- Most preferred	2	3	4	5	6- Least preferred	Should not be used
Intensity (e.g., sign/symptom level on a numeric rating scale)	○	○	○	○	○	○	○
Duration (e.g., hours, days, etc.)	○	○	○	○	○	○	○
Reversibility (e.g., reversible, irreversible)	○	○	○	○	○	○	○
Impact on a patient (e.g., quality of life, activities of daily living, etc.)	○	○	○	○	○	○	○
Additional care required (e.g., self care, additional healthcare, etc.)	○	○	○	○	○	○	○
Other	○	○	○	○	○	○	○

Please, specify: __________________________________

Intensity

Below are words used by participants in round 1 to describe the intensity of each category. Which word do you think best describes each severity category?

	Minimal	Low	Medium	High	Important	Significant
Benign	○	○	○	○	○	○
Mild	○	○	○	○	○	○
Minor	○	○	○	○	○	○
Moderate	○	○	○	○	○	○
Catastrophic	○	○	○	○	○	○
Major	○	○	○	○	○	○
Severe	○	○	○	○	○	○
Serious	○	○	○	○	○	○

On a generic 11-point numeric scale (0-11 points) where:
'0' = no sign/symptom
'10' = the worst sign/symptom
What are the minimum and maximum absolute values you think best describe each severity category:

Example- If I think the term 'Not bad' would have a minimum and maximum from 1-3 on this scale, I would answer:
Not bad - Minimum: 1
Not bad - Maximum, must be greater than 1: 3

	0	1	2	3	4	5	6	7	8	9	10
Benign - Minimum	○	○	○	○	○	○	○	○	○	○	○
Benign - Maximum, must be greater than [ben_abs_nrs_min].	○	○	○	○	○	○	○	○	○	○	○
Mild - Minimum	○	○	○	○	○	○	○	○	○	○	○
Mild - Maximum, must be greater than [mil_abs_nrs_min].	○	○	○	○	○	○	○	○	○	○	○
Minor - Minimum	○	○	○	○	○	○	○	○	○	○	○
Minor - Maximum, must be greater than [min_abs_nrs_min].	○	○	○	○	○	○	○	○	○	○	○
Moderate - Minimum	○	○	○	○	○	○	○	○	○	○	○
Moderate - Maximum, must be greater than [mod_abs_nrs_min].	○	○	○	○	○	○	○	○	○	○	○
Catastrophic - Minimum	○	○	○	○	○	○	○	○	○	○	○
Catastrophic - Maximum, must be greater than [cat_abs_nrs_min].	○	○	○	○	○	○	○	○	○	○	○
Major - Minimum	○	○	○	○	○	○	○	○	○	○	○
Major - Maximum, must be greater than [maj_abs_nrs_min].	○	○	○	○	○	○	○	○	○	○	○
Severe - Minimum	○	○	○	○	○	○	○	○	○	○	○
Severe - Maximum, must be greater than [sev_abs_nrs_min].	○	○	○	○	○	○	○	○	○	○	○
Serious - Minimum	○	○	○	○	○	○	○	○	○	○	○
Serious - Maximum, must be greater than [ser_abs_nrs_min].	○	○	○	○	○	○	○	○	○	○	○

In terms of relative increase from a patient's pre-intervention score on a generic 11-point numeric scale (0-11 points) where:
'0' = no increase in sign/symptom
'10' = the greatest possible increase in sign/symptom
What are the minimum and maximum values you think best describe the increase for each severity category:

Example- If I think the term 'Not bad' would have a minimum and maximum increase of 1-3 points on this scale (i.e., from a score of 5 to 6-8), I would answer:
Not bad - Minimum: 1
Not bad - Maximum, must be greater than 1: 3

	0	1	2	3	4	5	6	7	8	9	10
Benign - Minimum	○	○	○	○	○	○	○	○	○	○	○
Benign - Maximum, must be greater than [ben_abs_nrs_min].	○	○	○	○	○	○	○	○	○	○	○
Mild - Minimum	○	○	○	○	○	○	○	○	○	○	○
Mild - Maximum, must be greater than [mil_abs_nrs_min].	○	○	○	○	○	○	○	○	○	○	○
Minor - Minimum	○	○	○	○	○	○	○	○	○	○	○
Minor - Maximum, must be greater than [min_abs_nrs_min].	○	○	○	○	○	○	○	○	○	○	○
Moderate - Minimum	○	○	○	○	○	○	○	○	○	○	○
Moderate - Maximum, must be greater than [mod_abs_nrs_min].	○	○	○	○	○	○	○	○	○	○	○
Catastrophic - Minimum	○	○	○	○	○	○	○	○	○	○	○
Catastrophic - Maximum, must be greater than [cat_abs_nrs_min].	○	○	○	○	○	○	○	○	○	○	○
Major - Minimum	○	○	○	○	○	○	○	○	○	○	○
Major - Maximum, must be greater than [maj_abs_nrs_min].	○	○	○	○	○	○	○	○	○	○	○
Severe - Minimum	○	○	○	○	○	○	○	○	○	○	○
Severe - Maximum, must be greater than [sev_abs_nrs_min].	○	○	○	○	○	○	○	○	○	○	○
Serious - Minimum	○	○	○	○	○	○	○	○	○	○	○
Serious - Maximum, must be greater than [ser_abs_nrs_min].	○	○	○	○	○	○	○	○	○	○	○

Intensity preferences

What is your preferred option to best describe severity intensity?	◯ Wording (i.e., minimal, low, medium, high, important, significant)<br>◯ Number (i.e., minimum and maximum intensity)
What is your preferred number option to best describe severity intensity?	◯ Absolute values<br>◯ Relative increase

Duration

Below are words used by participants in round 1 to describe the duration of each category. Which option do you think best describes each severity category?

	Short term	Medium term	Long term
Benign	◯	◯	◯
Mild	◯	◯	◯
Minor	◯	◯	◯
Moderate	◯	◯	◯
Catastrophic	◯	◯	◯
Major	◯	◯	◯
Severe	◯	◯	◯
Serious	◯	◯	◯

What is the minimum and maximum duration that would best describe benign?

For benign, what is your preferred time allocation for the minimum duration:	◯ hour(s)<br>◯ day(s)<br>◯ week(s)<br>◯ month(s)<br>◯ year(s)
Benign - minimum duration:	◯ 1 hour<br>◯ 2 hours<br>◯ 3 hours<br>◯ 6 hours<br>◯ 12 hours<br>◯ 24 hours
Benign - minimum duration:	◯ 1 day<br>◯ 2 days<br>◯ 3 days<br>◯ 4 days<br>◯ 5 days<br>◯ 6 days<br>◯ 7 days
Benign - minimum duration:	◯ 1 week<br>◯ 2 weeks<br>◯ 3 weeks<br>◯ 4 weeks
Benign - minimum duration:	◯ 1 month<br>◯ 2 months<br>◯ 3 months<br>◯ 4 months<br>◯ 5 months<br>◯ 6 months<br>◯ 7 months<br>◯ 8 months<br>◯ 9 months<br>◯ 10 months<br>◯ 11 months<br>◯ 12 months
Benign - minimum duration:	◯ 1 year<br>◯ 2 years<br>◯ 3 years<br>◯ 4 years<br>◯ 5 years<br>◯ 10 years<br>◯ > 10 years
For benign, what is your preferred time allocation for the maximum duration:	◯ hour(s)<br>◯ day(s)<br>◯ week(s)<br>◯ month(s)<br>◯ year(s)
Benign - maximum duration:	◯ 1 hour<br>◯ 2 hours<br>◯ 3 hours<br>◯ 6 hours<br>◯ 12 hours<br>◯ 24 hours

Benign - maximum duration:	◯ 1 day<br>◯ 2 days<br>◯ 3 days<br>◯ 4 days<br>◯ 5 days<br>◯ 6 days<br>◯ 7 days
Benign - maximum duration:	◯ 1 week<br>◯ 2 weeks<br>◯ 3 weeks<br>◯ 4 weeks
Benign - maximum duration:	◯ 1 month<br>◯ 2 months<br>◯ 3 months<br>◯ 4 months<br>◯ 5 months<br>◯ 6 months<br>◯ 7 months<br>◯ 8 months<br>◯ 9 months<br>◯ 10 months<br>◯ 11 months<br>◯ 12 months
Benign - maximum duration:	◯ 1 year<br>◯ 2 years<br>◯ 3 years<br>◯ 4 years<br>◯ 5 years<br>◯ 10 years<br>◯ > 10 years

What is the minimum and maximum duration that would best describe mild?

For mild, what is your preferred time allocation for the minimum duration:
- ◯ hour(s)
- ◯ day(s)
- ◯ week(s)
- ◯ month(s)
- ◯ year(s)

Mild - minimum duration:
- ◯ 1 hour
- ◯ 2 hours
- ◯ 3 hours
- ◯ 6 hours
- ◯ 12 hours
- ◯ 24 hours

Mild - minimum duration:
- ◯ 1 day
- ◯ 2 days
- ◯ 3 days
- ◯ 4 days
- ◯ 5 days
- ◯ 6 days
- ◯ 7 days

Mild - minimum duration:
- ◯ 1 week
- ◯ 2 weeks
- ◯ 3 weeks
- ◯ 4 weeks

Mild - minimum duration:
- ◯ 1 month
- ◯ 2 months
- ◯ 3 months
- ◯ 4 months
- ◯ 5 months
- ◯ 6 months
- ◯ 7 months
- ◯ 8 months
- ◯ 9 months
- ◯ 10 months
- ◯ 11 months
- ◯ 12 months

Mild - minimum duration:
- ◯ 1 year
- ◯ 2 years
- ◯ 3 years
- ◯ 4 years
- ◯ 5 years
- ◯ 10 years
- ◯ > 10 years

For mild, what is your preferred time allocation for the maximum duration:
- ◯ hour(s)
- ◯ day(s)
- ◯ week(s)
- ◯ month(s)
- ◯ year(s)

Mild - maximum duration:
- ◯ 1 hour
- ◯ 2 hours
- ◯ 3 hours
- ◯ 6 hours
- ◯ 12 hours
- ◯ 24 hours

Mild - maximum duration:	◯ 1 day<br>◯ 2 days<br>◯ 3 days<br>◯ 4 days<br>◯ 5 days<br>◯ 6 days<br>◯ 7 days
Mild - maximum duration:	◯ 1 week<br>◯ 2 weeks<br>◯ 3 weeks<br>◯ 4 weeks
Mild - maximum duration:	◯ 1 month<br>◯ 2 months<br>◯ 3 months<br>◯ 4 months<br>◯ 5 months<br>◯ 6 months<br>◯ 7 months<br>◯ 8 months<br>◯ 9 months<br>◯ 10 months<br>◯ 11 months<br>◯ 12 months
Mild - maximum duration:	◯ 1 year<br>◯ 2 years<br>◯ 3 years<br>◯ 4 years<br>◯ 5 years<br>◯ 10 years<br>◯ > 10 years

What is the minimum and maximum duration that would best describe minor?

For minor, what is your preferred time allocation for the minimum duration:	◯ hour(s)<br>◯ day(s)<br>◯ week(s)<br>◯ month(s)<br>◯ year(s)
Minor - minimum duration:	◯ 1 hour<br>◯ 2 hours<br>◯ 3 hours<br>◯ 6 hours<br>◯ 12 hours<br>◯ 24 hours
Minor - minimum duration:	◯ 1 day<br>◯ 2 days<br>◯ 3 days<br>◯ 4 days<br>◯ 5 days<br>◯ 6 days<br>◯ 7 days
Minor - minimum duration:	◯ 1 week<br>◯ 2 weeks<br>◯ 3 weeks<br>◯ 4 weeks
Minor - minimum duration:	◯ 1 month<br>◯ 2 months<br>◯ 3 months<br>◯ 4 months<br>◯ 5 months<br>◯ 6 months<br>◯ 7 months<br>◯ 8 months<br>◯ 9 months<br>◯ 10 months<br>◯ 11 months<br>◯ 12 months
Minor - minimum duration:	◯ 1 year<br>◯ 2 years<br>◯ 3 years<br>◯ 4 years<br>◯ 5 years<br>◯ 10 years<br>◯ > 10 years
For minor, what is your preferred time allocation for the maximum duration:	◯ hour(s)<br>◯ day(s)<br>◯ week(s)<br>◯ month(s)<br>◯ year(s)
Minor - maximum duration:	◯ 1 hour<br>◯ 2 hours<br>◯ 3 hours<br>◯ 6 hours<br>◯ 12 hours<br>◯ 24 hours

Minor - maximum duration:	◯ 1 day<br>◯ 2 days<br>◯ 3 days<br>◯ 4 days<br>◯ 5 days<br>◯ 6 days<br>◯ 7 days
Minor - maximum duration:	◯ 1 week<br>◯ 2 weeks<br>◯ 3 weeks<br>◯ 4 weeks
Minor - maximum duration:	◯ 1 month<br>◯ 2 months<br>◯ 3 months<br>◯ 4 months<br>◯ 5 months<br>◯ 6 months<br>◯ 7 months<br>◯ 8 months<br>◯ 9 months<br>◯ 10 months<br>◯ 11 months<br>◯ 12 months
Minor - maximum duration:	◯ 1 year<br>◯ 2 years<br>◯ 3 years<br>◯ 4 years<br>◯ 5 years<br>◯ 10 years<br>◯ > 10 years

What is the minimum and maximum duration that would best describe moderate?

For moderate, what is your preferred time allocation for the minimum duration:	◯ hour(s)<br>◯ day(s)<br>◯ week(s)<br>◯ month(s)<br>◯ year(s)
Moderate - minimum duration:	◯ 1 hour<br>◯ 2 hours<br>◯ 3 hours<br>◯ 6 hours<br>◯ 12 hours<br>◯ 24 hours
Moderate - minimum duration:	◯ 1 day<br>◯ 2 days<br>◯ 3 days<br>◯ 4 days<br>◯ 5 days<br>◯ 6 days<br>◯ 7 days
Moderate - minimum duration:	◯ 1 week<br>◯ 2 weeks<br>◯ 3 weeks<br>◯ 4 weeks
Moderate - minimum duration:	◯ 1 month<br>◯ 2 months<br>◯ 3 months<br>◯ 4 months<br>◯ 5 months<br>◯ 6 months<br>◯ 7 months<br>◯ 8 months<br>◯ 9 months<br>◯ 10 months<br>◯ 11 months<br>◯ 12 months
Moderate - minimum duration:	◯ 1 year<br>◯ 2 years<br>◯ 3 years<br>◯ 4 years<br>◯ 5 years<br>◯ 10 years<br>◯ > 10 years
For moderate, what is your preferred time allocation for the maximum duration:	◯ hour(s)<br>◯ day(s)<br>◯ week(s)<br>◯ month(s)<br>◯ year(s)
Moderate - maximum duration:	◯ 1 hour<br>◯ 2 hours<br>◯ 3 hours<br>◯ 6 hours<br>◯ 12 hours<br>◯ 24 hours

Moderate - maximum duration:	◯ 1 day<br>◯ 2 days<br>◯ 3 days<br>◯ 4 days<br>◯ 5 days<br>◯ 6 days<br>◯ 7 days
Moderate - maximum duration:	◯ 1 week<br>◯ 2 weeks<br>◯ 3 weeks<br>◯ 4 weeks
Moderate - maximum duration:	◯ 1 month<br>◯ 2 months<br>◯ 3 months<br>◯ 4 months<br>◯ 5 months<br>◯ 6 months<br>◯ 7 months<br>◯ 8 months<br>◯ 9 months<br>◯ 10 months<br>◯ 11 months<br>◯ 12 months
Moderate - maximum duration:	◯ 1 year<br>◯ 2 years<br>◯ 3 years<br>◯ 4 years<br>◯ 5 years<br>◯ 10 years<br>◯ > 10 years

What is the minimum and maximum duration that would best describe catastrophic?

For catastrophic, what is your preferred time allocation for the minimum duration:

- ◯ hour(s)
- ◯ day(s)
- ◯ week(s)
- ◯ month(s)
- ◯ year(s)

Catastrophic - minimum duration:

- ◯ 1 hour
- ◯ 2 hours
- ◯ 3 hours
- ◯ 6 hours
- ◯ 12 hours
- ◯ 24 hours

Catastrophic - minimum duration:

- ◯ 1 day
- ◯ 2 days
- ◯ 3 days
- ◯ 4 days
- ◯ 5 days
- ◯ 6 days
- ◯ 7 days

Catastrophic - minimum duration:

- ◯ 1 week
- ◯ 2 weeks
- ◯ 3 weeks
- ◯ 4 weeks

Catastrophic - minimum duration:

- ◯ 1 month
- ◯ 2 months
- ◯ 3 months
- ◯ 4 months
- ◯ 5 months
- ◯ 6 months
- ◯ 7 months
- ◯ 8 months
- ◯ 9 months
- ◯ 10 months
- ◯ 11 months
- ◯ 12 months

Catastrophic - minimum duration:

- ◯ 1 year
- ◯ 2 years
- ◯ 3 years
- ◯ 4 years
- ◯ 5 years
- ◯ 10 years
- ◯ > 10 years

For catastrophic, what is your preferred time allocation for the maximum duration:

- ◯ hour(s)
- ◯ day(s)
- ◯ week(s)
- ◯ month(s)
- ◯ year(s)

Catastrophic - maximum duration:

- ◯ 1 hour
- ◯ 2 hours
- ◯ 3 hours
- ◯ 6 hours
- ◯ 12 hours
- ◯ 24 hours

Catastrophic - maximum duration:	◯ 1 day<br>◯ 2 days<br>◯ 3 days<br>◯ 4 days<br>◯ 5 days<br>◯ 6 days<br>◯ 7 days
Catastrophic - maximum duration:	◯ 1 week<br>◯ 2 weeks<br>◯ 3 weeks<br>◯ 4 weeks
Catastrophic - maximum duration:	◯ 1 month<br>◯ 2 months<br>◯ 3 months<br>◯ 4 months<br>◯ 5 months<br>◯ 6 months<br>◯ 7 months<br>◯ 8 months<br>◯ 9 months<br>◯ 10 months<br>◯ 11 months<br>◯ 12 months
Catastrophic - maximum duration:	◯ 1 year<br>◯ 2 years<br>◯ 3 years<br>◯ 4 years<br>◯ 5 years<br>◯ 10 years<br>◯ > 10 years

What is the minimum and maximum duration that would best describe major?

For major, what is your preferred time allocation for the minimum duration:	◯ hour(s)<br>◯ day(s)<br>◯ week(s)<br>◯ month(s)<br>◯ year(s)
Major - minimum duration:	◯ 1 hour<br>◯ 2 hours<br>◯ 3 hours<br>◯ 6 hours<br>◯ 12 hours<br>◯ 24 hours
Major - minimum duration:	◯ 1 day<br>◯ 2 days<br>◯ 3 days<br>◯ 4 days<br>◯ 5 days<br>◯ 6 days<br>◯ 7 days
Major - minimum duration:	◯ 1 week<br>◯ 2 weeks<br>◯ 3 weeks<br>◯ 4 weeks
Major - minimum duration:	◯ 1 month<br>◯ 2 months<br>◯ 3 months<br>◯ 4 months<br>◯ 5 months<br>◯ 6 months<br>◯ 7 months<br>◯ 8 months<br>◯ 9 months<br>◯ 10 months<br>◯ 11 months<br>◯ 12 months
Major - minimum duration:	◯ 1 year<br>◯ 2 years<br>◯ 3 years<br>◯ 4 years<br>◯ 5 years<br>◯ 10 years<br>◯ > 10 years
For major, what is your preferred time allocation for the maximum duration:	◯ hour(s)<br>◯ day(s)<br>◯ week(s)<br>◯ month(s)<br>◯ year(s)
Major - maximum duration:	◯ 1 hour<br>◯ 2 hours<br>◯ 3 hours<br>◯ 6 hours<br>◯ 12 hours<br>◯ 24 hours

Major - maximum duration:	◯ 1 day<br>◯ 2 days<br>◯ 3 days<br>◯ 4 days<br>◯ 5 days<br>◯ 6 days<br>◯ 7 days
Major - maximum duration:	◯ 1 week<br>◯ 2 weeks<br>◯ 3 weeks<br>◯ 4 weeks
Major - maximum duration:	◯ 1 month<br>◯ 2 months<br>◯ 3 months<br>◯ 4 months<br>◯ 5 months<br>◯ 6 months<br>◯ 7 months<br>◯ 8 months<br>◯ 9 months<br>◯ 10 months<br>◯ 11 months<br>◯ 12 months
Major - maximum duration:	◯ 1 year<br>◯ 2 years<br>◯ 3 years<br>◯ 4 years<br>◯ 5 years<br>◯ 10 years<br>◯ > 10 years

What is the minimum and maximum duration that would best describe severe?

For severe, what is your preferred time allocation for the minimum duration:	◯ hour(s)<br>◯ day(s)<br>◯ week(s)<br>◯ month(s)<br>◯ year(s)
Severe - minimum duration:	◯ 1 hour<br>◯ 2 hours<br>◯ 3 hours<br>◯ 6 hours<br>◯ 12 hours<br>◯ 24 hours
Severe - minimum duration:	◯ 1 day<br>◯ 2 days<br>◯ 3 days<br>◯ 4 days<br>◯ 5 days<br>◯ 6 days<br>◯ 7 days
Severe - minimum duration:	◯ 1 week<br>◯ 2 weeks<br>◯ 3 weeks<br>◯ 4 weeks
Severe - minimum duration:	◯ 1 month<br>◯ 2 months<br>◯ 3 months<br>◯ 4 months<br>◯ 5 months<br>◯ 6 months<br>◯ 7 months<br>◯ 8 months<br>◯ 9 months<br>◯ 10 months<br>◯ 11 months<br>◯ 12 months
Severe - minimum duration:	◯ 1 year<br>◯ 2 years<br>◯ 3 years<br>◯ 4 years<br>◯ 5 years<br>◯ 10 years<br>◯ > 10 years
For severe, what is your preferred time allocation for the maximum duration:	◯ hour(s)<br>◯ day(s)<br>◯ week(s)<br>◯ month(s)<br>◯ year(s)
Severe - maximum duration:	◯ 1 hour<br>◯ 2 hours<br>◯ 3 hours<br>◯ 6 hours<br>◯ 12 hours<br>◯ 24 hours

Severe - maximum duration:	◯ 1 day<br>◯ 2 days<br>◯ 3 days<br>◯ 4 days<br>◯ 5 days<br>◯ 6 days<br>◯ 7 days
Severe - maximum duration:	◯ 1 week<br>◯ 2 weeks<br>◯ 3 weeks<br>◯ 4 weeks
Severe - maximum duration:	◯ 1 month<br>◯ 2 months<br>◯ 3 months<br>◯ 4 months<br>◯ 5 months<br>◯ 6 months<br>◯ 7 months<br>◯ 8 months<br>◯ 9 months<br>◯ 10 months<br>◯ 11 months<br>◯ 12 months
Severe - maximum duration:	◯ 1 year<br>◯ 2 years<br>◯ 3 years<br>◯ 4 years<br>◯ 5 years<br>◯ 10 years<br>◯ > 10 years

What is the minimum and maximum duration that would best describe serious?

For serious, what is your preferred time allocation for the minimum duration:	◯ hour(s)<br>◯ day(s)<br>◯ week(s)<br>◯ month(s)<br>◯ year(s)
Serious - minimum duration:	◯ 1 hour<br>◯ 2 hours<br>◯ 3 hours<br>◯ 6 hours<br>◯ 12 hours<br>◯ 24 hours
Serious - minimum duration:	◯ 1 day<br>◯ 2 days<br>◯ 3 days<br>◯ 4 days<br>◯ 5 days<br>◯ 6 days<br>◯ 7 days
Serious - minimum duration:	◯ 1 week<br>◯ 2 weeks<br>◯ 3 weeks<br>◯ 4 weeks
Serious - minimum duration:	◯ 1 month<br>◯ 2 months<br>◯ 3 months<br>◯ 4 months<br>◯ 5 months<br>◯ 6 months<br>◯ 7 months<br>◯ 8 months<br>◯ 9 months<br>◯ 10 months<br>◯ 11 months<br>◯ 12 months
Serious - minimum duration:	◯ 1 year<br>◯ 2 years<br>◯ 3 years<br>◯ 4 years<br>◯ 5 years<br>◯ 10 years<br>◯ > 10 years
For serious, what is your preferred time allocation for the maximum duration:	◯ hour(s)<br>◯ day(s)<br>◯ week(s)<br>◯ month(s)<br>◯ year(s)
Serious - maximum duration:	◯ 1 hour<br>◯ 2 hours<br>◯ 3 hours<br>◯ 6 hours<br>◯ 12 hours<br>◯ 24 hours

Serious - maximum duration:	◯ 1 day<br>◯ 2 days<br>◯ 3 days<br>◯ 4 days<br>◯ 5 days<br>◯ 6 days<br>◯ 7 days
Serious - maximum duration:	◯ 1 week<br>◯ 2 weeks<br>◯ 3 weeks<br>◯ 4 weeks
Serious - maximum duration:	◯ 1 month<br>◯ 2 months<br>◯ 3 months<br>◯ 4 months<br>◯ 5 months<br>◯ 6 months<br>◯ 7 months<br>◯ 8 months<br>◯ 9 months<br>◯ 10 months<br>◯ 11 months<br>◯ 12 months
Serious - maximum duration:	◯ 1 year<br>◯ 2 years<br>◯ 3 years<br>◯ 4 years<br>◯ 5 years<br>◯ 10 years<br>◯ > 10 years

Duration preference

What is your preferred option to best describe duration?

- ◯ Wording (i.e., short, medium, long term)
- ◯ Number (i.e., minimum and maximum time)

Reversibility
Indicate if you think adverse events within each severity category are "reversible" or "irreversible":

	Reversible	Irreversible
Benign	◯	◯
Mild	◯	◯
Minor	◯	◯
Moderate	◯	◯
Catastrophic	◯	◯
Major	◯	◯
Severe	◯	◯
Serious	◯	◯

Below are words used by participants in round 1 to describe the reversibility of each category.

Please rank your preferred terms:

	1-Most preferred	2	3	4-Least preferred
Reversible	◯	◯	◯	◯
Short lasting	◯	◯	◯	◯
Temporary	◯	◯	◯	◯
Transient	◯	◯	◯	◯

Please rank your preferred terms:

	1-Most preferred	2	3	4-Least preferred
Irreversible	○	○	○	○
Long lasting	○	○	○	○
Permanent	○	○	○	○
Persistent	○	○	○	○

Impact on a patient
Please read and apply the following operational definitions for the questions below:[1]

Participation: Involvement in a life situation
Participation restrictions: Problems an individual may experience in involvement in life situations
Activity: The execution of a task or action by an individual
Activity limitations: Difficulties an individual may have in executing activities

[1]- The ICF: An Overview. Available at: https://www.wcpt.org/sites/wcpt.org/files/files/GH-ICF_overview_FINAL_for_WHO.pdf [accessed on 09 Mar 2022]

Below are words used by participants in round 1 to describe the impact on a patient of each category.

Which areas do you think best describe the impact of each severity category?

	No impact	Quality of life / participation	Activities / disability	Life threatening	Death
Benign	☐	☐	☐	☐	☐
Mild	☐	☐	☐	☐	☐
Minor	☐	☐	☐	☐	☐
Moderate	☐	☐	☐	☐	☐
Catastrophic	☐	☐	☐	☐	☐
Major	☐	☐	☐	☐	☐
Severe	☐	☐	☐	☐	☐
Serious	☐	☐	☐	☐	☐

Use the following modified scale to rate the extent of identified impairment, activity limitation or participation restriction:[1]

1 Tolerable
2 Some interference in day-to-day life
3 Considerable disruption of day-to-day life
4 Total disruption of day-to-day life
[1]- The ICF: An Overview. Available at: https://www.wcpt.org/sites/wcpt.org/files/files/GH-ICF_overview_FINAL_for_WHO.pdf [accessed on 09 Mar 2022]

Indicate the impact on quality of life/participation for benign:

- ◯ Tolerable
- ◯ Some interference in day-to-day quality of life/participation
- ◯ Considerable disruption of day-to-day quality of life/participation
- ◯ Total disruption of day-to-day quality of life/participation

Indicate the impact on quality of life/participation for mild:	◯ Tolerable<br>◯ Some interference in day-to-day quality of life/participation<br>◯ Considerable disruption of day-to-day quality of life/participation<br>◯ Total disruption of day-to-day quality of life/participation
Indicate the impact on quality of life/participation for minor:	◯ Tolerable<br>◯ Some interference in day-to-day quality of life/participation<br>◯ Considerable disruption of day-to-day quality of life/participation<br>◯ Total disruption of day-to-day quality of life/participation
Indicate the impact on quality of life/participation for moderate:	◯ Tolerable<br>◯ Some interference in day-to-day quality of life/participation<br>◯ Considerable disruption of day-to-day quality of life/participation<br>◯ Total disruption of day-to-day quality of life/participation
Indicate the impact on quality of life/participation for catastrophic:	◯ Tolerable<br>◯ Some interference in day-to-day quality of life/participation<br>◯ Considerable disruption of day-to-day quality of life/participation<br>◯ Total disruption of day-to-day quality of life/participation
Indicate the impact on quality of life/participation for major:	◯ Tolerable<br>◯ Some interference in day-to-day quality of life/participation<br>◯ Considerable disruption of day-to-day quality of life/participation<br>◯ Total disruption of day-to-day quality of life/participation
Indicate the impact on quality of life/participation for severe:	◯ Tolerable<br>◯ Some interference in day-to-day quality of life/participation<br>◯ Considerable disruption of day-to-day quality of life/participation<br>◯ Total disruption of day-to-day quality of life/participation
Indicate the impact on quality of life/participation for serious:	◯ Tolerable<br>◯ Some interference in day-to-day quality of life/participation<br>◯ Considerable disruption of day-to-day quality of life/participation<br>◯ Total disruption of day-to-day quality of life/participation
Indicate the impact on activity/disability for benign:	◯ Tolerable<br>◯ Some interference in day-to-day life activity<br>◯ Considerable disruption of day-to-day life activity<br>◯ Total disruption of day-to-day life activity

Indicate the impact on activity/disability for mild:	◯ Tolerable<br>◯ Some interference in day-to-day life activity<br>◯ Considerable disruption of day-to-day life activity<br>◯ Total disruption of day-to-day life activity
Indicate the impact on activity/disability for minor:	◯ Tolerable<br>◯ Some interference in day-to-day life activity<br>◯ Considerable disruption of day-to-day life activity<br>◯ Total disruption of day-to-day life activity
Indicate the impact on activity/disability for moderate:	◯ Tolerable<br>◯ Some interference in day-to-day life activity<br>◯ Considerable disruption of day-to-day life activity<br>◯ Total disruption of day-to-day life activity
Indicate the impact on activity/disability for catastrophic:	◯ Tolerable<br>◯ Some interference in day-to-day life activity<br>◯ Considerable disruption of day-to-day life activity<br>◯ Total disruption of day-to-day life activity
Indicate the impact on activity/disability for major:	◯ Tolerable<br>◯ Some interference in day-to-day life activity<br>◯ Considerable disruption of day-to-day life activity<br>◯ Total disruption of day-to-day life activity
Indicate the impact on activity/disability for severe:	◯ Tolerable<br>◯ Some interference in day-to-day life activity<br>◯ Considerable disruption of day-to-day life activity<br>◯ Total disruption of day-to-day life activity
Indicate the impact on activity/disability for serious:	◯ Tolerable<br>◯ Some interference in day-to-day life activity<br>◯ Considerable disruption of day-to-day life activity<br>◯ Total disruption of day-to-day life activity

Additional care required
Below are types of care used by participants in round 1 to describe the additional care required within each category.

Self care: e.g., ice/heat, non-prescription medication
Additional care from same provider: e.g., monitoring/observation, advice, early follow-up, treatment alteration
Additional care from another healthcare provider: e.g., medical or non-medical health care evaluation/intervention

Which type of care do you think best describes the additional care required within each category?

	None required	Self care	Additional care from same provider	Additional care from another healthcare provider
Benign	○	○	○	○
Mild	○	○	○	○
Minor	○	○	○	○
Moderate	○	○	○	○
Catastrophic	○	○	○	○
Major	○	○	○	○
Severe	○	○	○	○
Serious	○	○	○	○

Indicate the additional care from another healthcare provider for benign:
○ Non-medical healthcare evaluation/intervention (e.g., nurse, pharmacist, manual therapist, etc.)
○ Medical care

Indicate the additional care from another healthcare provider for mild:
○ Non-medical healthcare evaluation/intervention (e.g., nurse, pharmacist, manual therapist, etc.)
○ Medical care

Indicate the additional care from another healthcare provider for minor:
○ Non-medical healthcare evaluation/intervention (e.g., nurse, pharmacist, manual therapist, etc.)
○ Medical care

Indicate the additional care from another healthcare provider for moderate:
○ Non-medical healthcare evaluation/intervention (e.g., nurse, pharmacist, manual therapist, etc.)
○ Medical care

Indicate the additional care from another healthcare provider for catastrophic:
○ Non-medical healthcare evaluation/intervention (e.g., nurse, pharmacist, manual therapist, etc.)
○ Medical care

Indicate the additional care from another healthcare provider for major:
○ Non-medical healthcare evaluation/intervention (e.g., nurse, pharmacist, manual therapist, etc.)
○ Medical care

Indicate the additional care from another healthcare provider for severe:	◯ Non-medical healthcare evaluation/intervention (e.g., nurse, pharmacist, manual therapist, etc.)<br>◯ Medical care
Indicate the additional care from another healthcare provider for serious:	◯ Non-medical healthcare evaluation/intervention (e.g., nurse, pharmacist, manual therapist, etc.)<br>◯ Medical care
Please specify urgency/emergency for benign:	◯ Urgent/emergency<br>◯ Non-urgent/non-emergency
Please specify urgency/emergency for mild:	◯ Urgent/emergency<br>◯ Non-urgent/non-emergency
Please specify urgency/emergency for minor:	◯ Urgent/emergency<br>◯ Non-urgent/non-emergency
Please specify urgency/emergency for moderate:	◯ Urgent/emergency<br>◯ Non-urgent/non-emergency
Please specify urgency/emergency for catastrophic:	◯ Urgent/emergency<br>◯ Non-urgent/non-emergency
Please specify urgency/emergency for major:	◯ Urgent/emergency<br>◯ Non-urgent/non-emergency
Please specify urgency/emergency for severe:	◯ Urgent/emergency<br>◯ Non-urgent/non-emergency
Please specify urgency/emergency for serious:	◯ Urgent/emergency<br>◯ Non-urgent/non-emergency
Please specify care for benign:	☐ Medical evaluation/intervention<br>☐ Hospitalization<br>☐ Surgery
Please specify care for mild:	☐ Medical evaluation/intervention<br>☐ Hospitalization<br>☐ Surgery
Please specify care for minor:	☐ Medical evaluation/intervention<br>☐ Hospitalization<br>☐ Surgery
Please specify care for moderate:	☐ Medical evaluation/intervention<br>☐ Hospitalization<br>☐ Surgery
Please specify care for catastrophic:	☐ Medical evaluation/intervention<br>☐ Hospitalization<br>☐ Surgery
Please specify care for major:	☐ Medical evaluation/intervention<br>☐ Hospitalization<br>☐ Surgery

Please specify care for severe:	☐ Medical evaluation/intervention<br>☐ Hospitalization<br>☐ Surgery
Please specify care for serious:	☐ Medical evaluation/intervention<br>☐ Hospitalization<br>☐ Surgery
Please specify care for benign:	☐ Medical evaluation/intervention<br>☐ Hospitalization<br>☐ Surgery
Please specify care for mild:	☐ Medical evaluation/intervention<br>☐ Hospitalization<br>☐ Surgery
Please specify care for minor:	☐ Medical evaluation/intervention<br>☐ Hospitalization<br>☐ Surgery
Please specify care for moderate:	☐ Medical evaluation/intervention<br>☐ Hospitalization<br>☐ Surgery
Please specify care for catastrophic:	☐ Medical evaluation/intervention<br>☐ Hospitalization<br>☐ Surgery
Please specify care for major:	☐ Medical evaluation/intervention<br>☐ Hospitalization<br>☐ Surgery
Please specify care for severe:	☐ Medical evaluation/intervention<br>☐ Hospitalization<br>☐ Surgery
Please specify care for serious:	☐ Medical evaluation/intervention<br>☐ Hospitalization<br>☐ Surgery

Comment section

Please provide any additional thoughts or comments regarding the severity classification for adverse events following spinal and/or peripheral joint manipulation and/or mobilization. ______________________________________

Thank you!

This is the end of the round 2 questionnaire. Thank you for your participation and continued contribution to this important project.

Round 2 is open until April 27th, 2022. We will analyse the results and develop a round 3 questionnaire using the responses you and other participants have provided. We will provide you with a link to the round 3 questionnaire about 3 weeks after round 2 is complete.

Best regards,

Martha Funabashi, Lindsay Gorrell, Katie Pohlman, Andrea Bergna, Nicola Heneghan

If you have any questions, please contact: Martha Funabashi (mfunabashi@cmcc.ca)

AE Delphi - Round 3

EXPERT CONSENSUS ON A STANDARDIZED DEFINITION AND SEVERITY CLASSIFICATION FOR ADVERSE EVENTS FOLLOWING SPINAL AND PERIPHERAL JOINT MANIPULATION AND MOBILIZATION: AN INTERNATIONAL E-DELPHI STUDY

ROUND 3 Investigators:
- Martha Funabashi, BSc, MSc, PhD - Canadian Memorial Chiropractic College (Canada)
- Lindsay M Gorrell, BChiroSc, MChiroprac, MRes, PhD - University of Zurich (Switzerland)
- Katherine A Pohlman, DC, MS, PhD - Parker University (USA)
- Andrea Bergna, BSc, PT, DO - SOMA Instituto Osteopatia Milano (Italy)
- Nicola R Heneghan, PhD, MSc, PG Cert HEd, Grad Dip Phys - University of Birmingham (England)

REMINDER of study terms:
Spinal and peripheral joint manipulation and mobilization are interventions commonly used by a variety of healthcare professionals (e.g. chiropractors, naprapaths, naturopaths, osteopaths, physiotherapists, etc.) in the management of many musculoskeletal conditions, including spinal pain, and are most often administered in ambulatory care settings. These interventions are described by a vast array of terms, including high-velocity low-amplitude manipulation, low-velocity variable-amplitude mobilization, spinal manipulative therapy, musculoskeletal manipulation/mobilization, etc.

In this study:

Manipulation (also called adjustment) refers to a treatment that involves the therapist applying one rapid force/thrust to a joint with the aim of relieving pain or stiffness; and

Mobilization refers to a treatment that involves the therapist applying a force repeatedly (at variable speeds and amplitudes) to a joint with the aim of relieving pain or stiffness.

Spinal refers to joints of the neck, mid and low back.

Peripheral joint refers to shoulders, arms, hands, fingers, hips, legs, feet and toes.

If, at any time during this questionnaire, you have any questions, please feel free to contact Martha Funabashi at mfunabashi@cmcc.ca.

Round 3 questions were developed based on data from round 1 (180 respondents), round 2 (163 respondents), and a scoping review of the literature (69 studies) related to the definition and severity classification for adverse events following spinal and/or peripheral joint manipulation and/or mobilization. The questions include terms that were used to define and classify the severity of adverse events following spinal and/or peripheral joint manipulation and/or mobilization.   Estimated time for completion: 10-15 minutes.

Feedback from Round 2 - Adverse Event Definition

Consensus regarding the definition for an adverse event:
89.5% agreed that an adverse event is undesired.
85.8% agreed that an adverse event is unfavourable.
84.0% agreed that an adverse event is unintended.
61.7% agreed that an adverse event is unexpected.
78.4% agreed that an adverse event can occur when no manipulation and/or mobilization is delivered.

72.2% disagreed that an adverse event is expected.
68.5% disagreed that the persistence of signs, symptoms or disease is an adverse event.
67.9% disagreed that an adverse event is unequivocally caused by spinal and/or peripheral joint manipulation and/or mobilization.

From open text comments, participant comments centred on the following:
- It is important to keep the definition as short as possible so it is easily communicated to and understood by patients.
- There are too many adjectives at the beginning of the definition and "unintended" should be removed as an adverse event is an adverse event regardless of intent.
- While the definition should specify the potential relationship between manipulation and/or mobilization and the adverse event, determining causality is not possible.
- The wording "whether or not caused by that manipulation and/or mobilization" should be removed from the proposed definition ("An adverse event is defined as any expected or unexpected, unfavourable, undesired and unintended outcome that occurs during or after spinal and/or peripheral joint manipulation and/or mobilization, whether or not caused by that manipulation and/or mobilization.").

The following questions relate to the definition for an adverse event following spinal and/or peripheral joint manipulation and/or mobilization.

Please rank the following statements indicating your preferred definition for an adverse event:

	Most Preferred		Least Preferred
An adverse event is any unfavourable outcome that occurs during or following spinal and/or peripheral manipulation and/or mobilization.	○	○	○
An adverse event is any unfavourable and unexpected outcome that occurs during or following spinal and/or peripheral manipulation and/or mobilization.	○	○	○
An adverse event is any unfavourable, unexpected and undesired outcome that occurs during or following spinal and/or peripheral manipulation and/or mobilization.	○	○	○

If you have any comments related to the adverse event definition , please provide them here.

____

Feedback from Round 2 - Severity Classification

Consensus regarding the severity classification for an adverse event:
86.1% agreed that "mild" and "minor" are similar severity categories.
79.0% stated a preference for the term "mild" compared to "minor".
67.7% agreed that "major" and "severe" are similar severity categories.
81.8% stated a preference for the term "severe" compared to "major".
87.3% agreed that the impact on a patient should be used to differentiate between severity categories.
75.9% agreed that intensity should be used to differentiate between severity categories.

From open text comments, participant comments centred mainly on the following:
- "Benign" should not be a severity category.
- It is difficult to differentiate between "major" and "severe" categories.
- "Serious adverse event" has an established definition within the wider health care field, and this existing definition should be used for manipulation and/or mobilization.
- "Duration" should not be used to differentiate between severity categories.

The following question relate to the severity classification for adverse events following spinal and/or peripheral joint manipulation and/or mobilization.

Please indicate your level of agreement with the statement:

"Benign" should not be used as an adverse event severity category as it relates to an outcome that is ''not adverse''.

◯ Strongly Agree ◯ Agree ◯ Neutral ◯ Disagree ◯ Strongly Disagree

If you have any comments related to "benign" adverse event, please provide them here.

____

For the next questions, please indicate your level of agreement with the following statements, regardless of your level of agreement with the "benign" statement.

	Strongly Agree	Agree	Neutral	Disagree	Strongly Disagree
A "benign" adverse event has minimal intensity, ranging between 0-1 on an 11-point numeric scale.	○	○	○	○	○
A "mild" adverse event has a low intensity, ranging between 1-3 on an 11-point numeric scale.	○	○	○	○	○
A "moderate" adverse event has a moderate intensity, ranging between 3-6 on an 11-point numeric scale.	○	○	○	○	○
A "severe" adverse event has a high intensity, ranging between 6-8 on an 11-point numeric scale.	○	○	○	○	○
A "catastrophic" adverse event has a significant intensity, ranging between 8-10 on an 11-point numeric scale.	○	○	○	○	○

If you have any comments related to the adverse event severity classification and intensity, please provide them here. ______________________________________

For the next statements, please consider the same operational definitions provided in Round 2:[1]
Activity: The execution of a task or action by an individual.
Activity Limitations: Difficulties an individual may have in executing activities.
Participation: Involvement in a life situation.
Participation Restrictions: Problems an individual may experience in involvement in life situations.[1]- The ICF: An Overview. Available at: https://www.wcpt.org/sites/wcpt.org/files/files/GH-ICF_overview_FINAL_for_WHO.pdf [accessed on 09 Mar 2022]
ADDITIONALLY - Quality of Life is defined as 'an individual's perception of their position in life in the context of the culture and value systems in which they live and in relation to their goals, expectations, standards and concerns'.[2][2]- World Health Organization (WHO) - WHOQOL: Measuring Quality of Life. Available at: https://www.who.int/toolkits/whoqol [accessed on 17 May 2022]

For the next questions, please indicate your level of agreement with the following statements, regardless of your level of agreement with the "benign" statement.

	Strongly Agree	Agree	Neutral	Disagree	Strongly Disagree
A "benign" adverse event has no impact on a patient's activities or quality of life.	○	○	○	○	○
A "mild" adverse event has no impact on a patient's activities, but it has a tolerable interference on participation, and quality of life.	○	○	○	○	○
A "moderate" adverse event has some interference with a patient's activities, participation, and quality of life.	○	○	○	○	○
A "severe" adverse event is not life-threatening, but has considerable interference with a patient's activities, participation, and quality of life.	○	○	○	○	○
A "catastrophic" adverse event is life-threatening and could result in death; it totally disrupts a patient's activities, participation, and quality of life.	○	○	○	○	○

If you have any comments related to the adverse event severity classification and impact on a patient, please provide them here. ________________________________

Serious and Catastrophic Adverse Events

A "serious adverse event" (SAE) has an established definition within the wider healthcare field, that is: "Any untoward medical occurrence that results in death, is life-threatening requires inpatient hospitalization or causes prolongation of existing hospitalization results in persistent or significant disability/incapacity, may have caused a congenital anomaly/birth defect, or requires intervention to prevent permanent impairment or damage."[3-7]

At this time, data from this study suggest that a "catastrophic" adverse event "has significant intensity, ranging between 8-10 on an 11-point numeric scale; is life-threatening and could result in death; it totally disrupts a patient's activities, participation, and quality of life."

Please indicate your level of agreement that these two definitions should be considered similar.

◯ Strongly Agree ◯ Agree ◯ Neutral ◯ Disagree ◯ Strongly Disagree

---

[3]- FDA (2009) Guidance for Clinical Investigators, Sponsors, and IRBs Adverse Event Reporting to IRBs - Improving Human Subject Protection;
[4]- ICH E2A: International Conference on Harmonisation - Tripartite Guideline: Clinical Safety Data Management: Definitions and Standards for Expedited Reporting (1994);
[5]- ICH E6 (R2): International Council for Harmonisation - Harmonised Guideline: Guideline for Good Clinical Practice (2016; reflects ICH name change);
[6]- Office for Human Subjects Protection, Division of Department of Health and Human Services Policy; "Unanticipated Problems Involving Risks and Adverse Events Guidance" (2007);
[7]- Code of Federal Regulations Title 21, Part 312.32 (2016)

If you have any comments related to serious and catastrophic adverse events, please provide them here. ______________________________

Focus Group Participation Interest

Would you be interested in participating in a focus group to further explore findings from this study?	◯ Yes ◯ No
Please provide your contact information:	__________________________________

Thank you!

This is the end of the round 3 questionnaire. Thank you for your participation and continued contribution to this important project.

Round 3 is open until June 30th, 2022. We will analyse the results and develop the final products with recommended next steps using the responses you and other participants have provided.

Best regards,

Martha Funabashi, Lindsay Gorrell, Katie Pohlman, Andrea Bergna, Nicola Heneghan

If you have any questions, please contact: Martha Funabashi (mfunabashi@cmcc.ca).
