# Supplementary material for: Defining and classifying adverse events following joint manipulation and mobilization: An international e-Delphi study and focus groups
Source: PLoS One. 2025 Nov 17;20(11):e0334151. doi: 10.1371/journal.pone.0334151 (PMC12622795; doi:10.1371/journal.pone.0334151)
Supplement: S2 File — (DOCX) [file pone.0334151.s007.docx]

**S2 File**

**Summary of e-Delphi results and discussion topics**

**Standardized definition and severity classification for adverse events following spinal and peripheral joint manipulation and mobilization**

**e-Delphi recap**

**OBJECTIVE:**

The objective of the e-Delphi study was to determine, by an expert consensus process, a standardised definition and severity classification for adverse events following spinal and peripheral joint manipulation and mobilization, within an adult population with musculoskeletal conditions, for use in both clinical care and research studies.

**METHODS:**

*Design:* Electronic (online) Delphi study

*Participants:* Experts within the following groups were recruited:


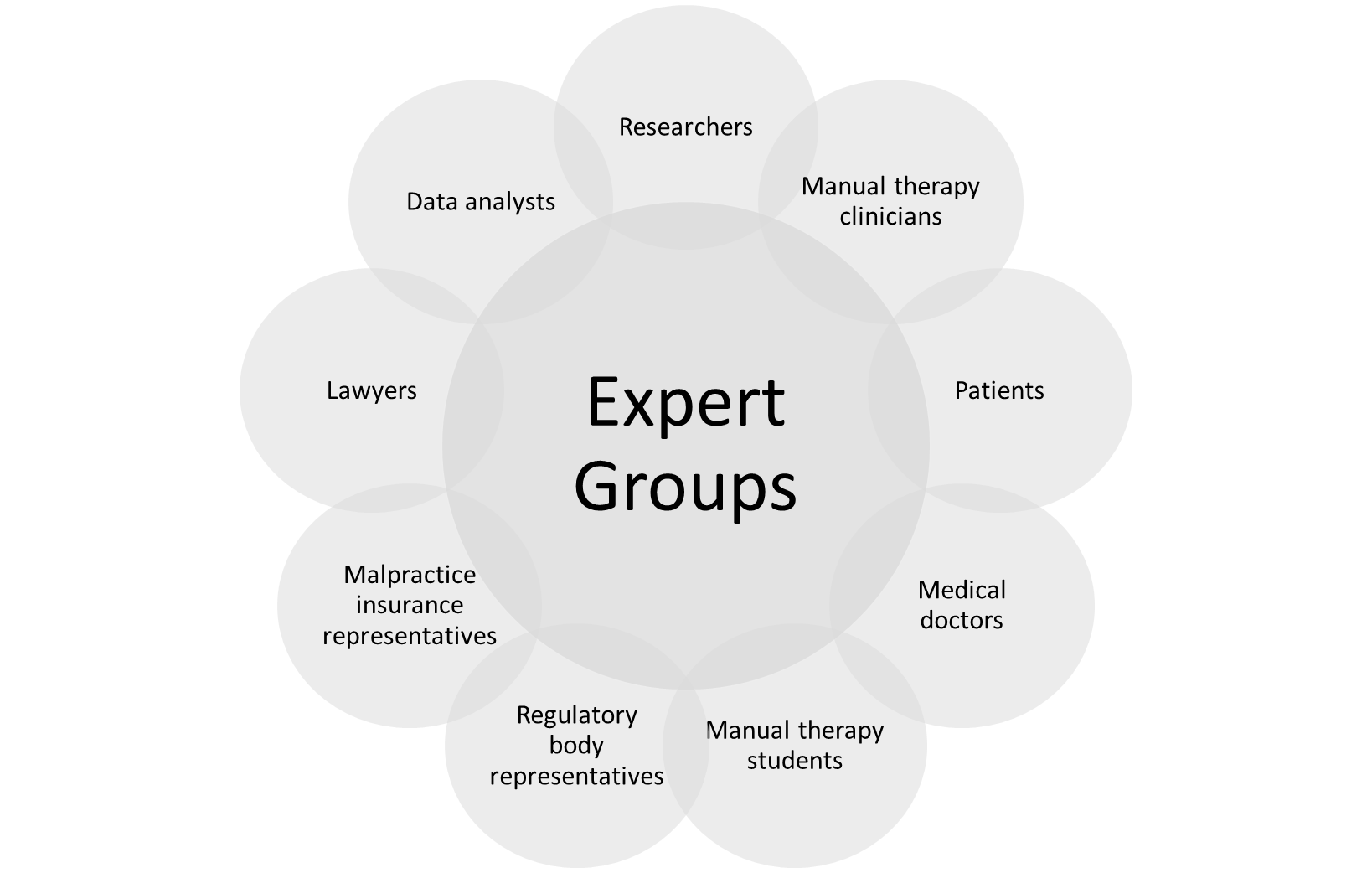


*Procedure:* Three rounds of questionnaires were sent to participants as described below:


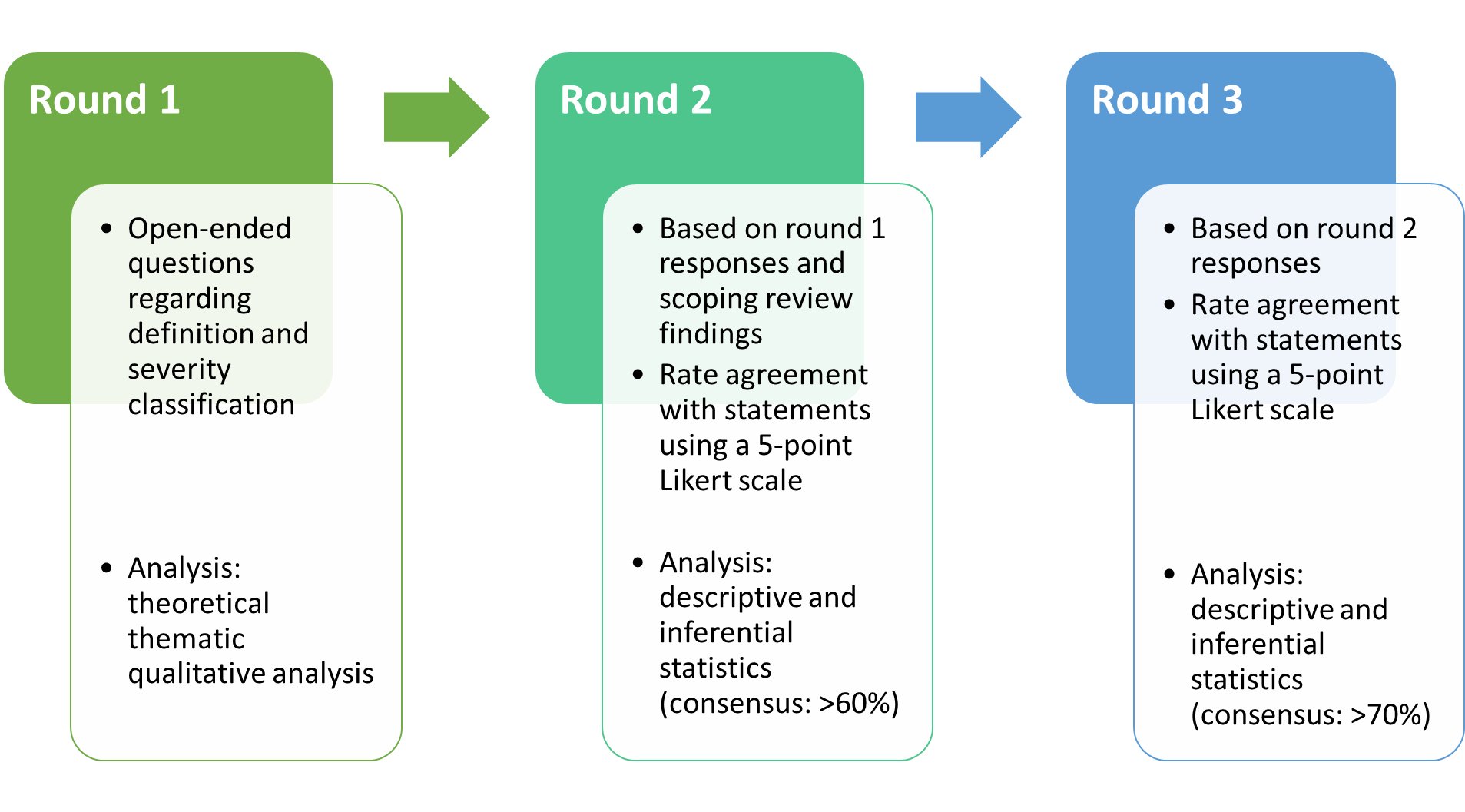


- All questions from all rounds were revised by an advisory committee
- All participants were invited to respond to all rounds
- All rounds were open for 6 weeks, with email reminders sent at weeks 1, 3 and 5

For additional details, please see full protocol [here](https://bmjopen.bmj.com/content/bmjopen/11/11/e050219.full.pdf?with-ds=yes).

**SUMMARY OF FINDINGS:**

Each of the three rounds was completed by over 150 respondents, with participant representation from all professions, expert groups and geographical locations (all continents).

At the end of round 3, there were two items that did not reach consensus:

1) the **definition** for “adverse events” following spinal and peripheral joint manipulation and mobilization; and

2) the **use of “serious” versus “catastrophic”** as categories for classifying adverse events.

1. *Definition:*

Based on the first two rounds, the following definitions were proposed in the third round with no consensus achieved:

- 1. An adverse event is any **unfavourable** outcome that occurs during or following spinal and/or peripheral manipulation and/or mobilization.
  2. An adverse event is any **unfavourable and unexpected** outcome that occurs during or following spinal and/or peripheral manipulation and/or mobilization.
  3. An adverse event is any **unfavourable, unexpected and undesired** outcome that occurs during or following spinal and/or peripheral manipulation and/or mobilization.

1. *Use of ''serious'' versus ''catastrophic'':*

A "serious adverse event" (SAE) has an established definition within the wider healthcare field, that is:

"Any untoward medical occurrence that results in death, is life-threatening requires inpatient hospitalization or causes prolongation of existing hospitalization results in persistent or significant disability/incapacity, may have caused a congenital anomaly/birth defect, or requires intervention to prevent permanent impairment or damage."

Data from this study suggested that a "catastrophic" adverse event "has significant intensity, ranging between 8-10 on an 11-point numeric scale; is life-threatening and could result in death; it totally disrupts a patient's activities, participation, and quality of life."

There was no consensus that these two categories and that the two definitions should be considered similar.
