# Supplementary figures and images for: Defining and classifying adverse events following joint manipulation and mobilization: An international e-Delphi study and focus groups
Source: PLoS One. 2025 Nov 17;20(11):e0334151. doi: 10.1371/journal.pone.0334151 (PMC12622795; doi:10.1371/journal.pone.0334151)

**S1 Fig**

**Representation of the main themes discussed during the focus groups**

**
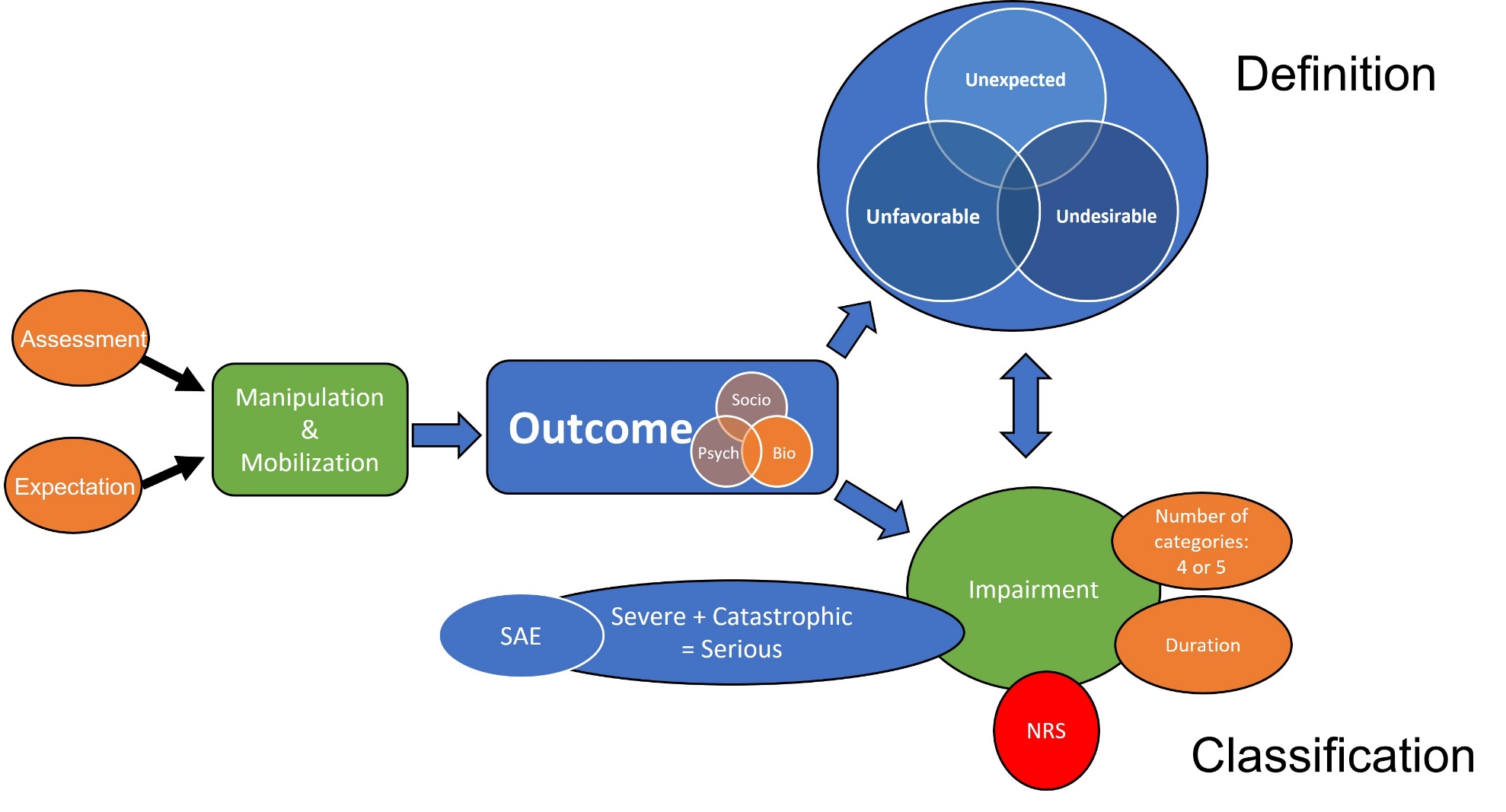
**

Supplement: S1 Fig — (DOCX) [file pone.0334151.s008.docx]
